# Egg components and offspring survival vary with group size and laying order in a cooperative breeder

**DOI:** 10.1007/s00442-023-05379-w

**Published:** 2023-05-06

**Authors:** Rita Fortuna, Matthieu Paquet, Clotilde Biard, Loïc Élard, André C. Ferreira, Mathieu Leroux-Coyaux, Charline Parenteau, Liliana R. Silva, Franck Théron, Rita Covas, Claire Doutrelant

**Affiliations:** 1 Present Address: CIBIO, Centro de Investigação em Biodiversidade e Recursos Genéticos, InBIO Laboratório Associado, Campus de Vairão, Universidade do Porto, 4485-661 Vairão, Portugal; 2 Departamento de Biologia, Faculdade de Ciências, Universidade do Porto, 4099-002 Porto, Portugal; 3 BIOPOLIS Program in Genomics, Biodiversity and Land Planning, CIBIO, Campus de Vairão, 4485-661 Vairão, Portugal; 4 Centre d’Ecologie Fonctionnelle et Evolutive, CEFE, CNRS, Univ Montpellier, EPHE, IRD, 34293 Montpellier, France; 5 Centre for Biodiversity Dynamics, Institutt for Biologi, NTNU, Trondheim, Norway; 6 Department of Ecology, Swedish University of Agricultural Sciences, P.O. Box 7044, 75007 Uppsala, Sweden; 7 Institute of Mathematics of Bordeaux, University of Bordeaux, CNRS, Bordeaux INP, Talence, France; 8 Sorbonne Université, UPEC, CNRS, IRD, INRA, Institut d’Écologie et des Sciences de l’Environnement de Paris, IEES, 75005 Paris, France; 9 Centre d’Etudes Biologiques de Chizé, CNRS-La Rochelle Université, Villiers-en-Bois, France; 10 DST-NRF Centre of Excellence, FitzPatrick Institute, University of Cape Town, Cape Town, South Africa; 11 Department of Evolutionary Biology and Environmental Studies, University of Zurich, Winterthurerstrasse 190, 8057 Zurich, Switzerland

**Keywords:** Cooperative breeding, Differential allocation, Helpers, Laying order, Load-lightening

## Abstract

Prenatal resource allocation to offspring can be influenced by maternal environment and offspring value, and affect offspring survival. An important pathway for flexible maternal allocation is via egg components such as nutrients and hormones. In cooperative breeders, females with helpers may increase resource allocation to eggs—‘differential allocation’—or reduce it—‘load-lightening’. Yet, helper effects on egg composition have been poorly studied. Moreover, it is unknown how helpers’ presence modulates laying order effects on egg content and survival. Here, we investigated how maternal allocation varied with group size and laying order in the cooperatively breeding sociable weaver (*Philetairus socius*). We estimated interactive effects of helpers and laying order on allocation to egg mass, yolk nutrients—yolk mass, proteins, lipids, carotenoids, vitamin A and vitamin E—and hormones—testosterone, androstenedione, and corticosterone. Results concurred with the ‘differential allocation’ predictions. Females with more helpers produced later-laid eggs with heavier yolks and more lipids, and laid eggs overall richer in lipids. Proteins, antioxidants, and hormones were not found to vary with helper number. We then analyzed how helper number modulated laying order effects on survival. Females with more helpers did not specifically produce later-laid eggs with higher survival, but eggs laid by females with more helpers were overall more likely to fledge. These findings show that some egg components (yolk mass, lipids) can positively vary according to females’ breeding group size, which may improve offspring fitness.

**Supplementary Information:**

The online version contains supplementary material available at 10.1007/s00442-023-05379-w.

## Introduction

Prenatal reproductive investment can vary with the breeding conditions experienced by females and may be adjusted to the expected fitness value of their current breeding event (Mousseau and Fox 1998). In oviparous species, the essential resources for embryonic development are accumulated in the eggs (Carey 1996). Mothers’ condition or experience can lead to variation in the quantity and quality of these resources, which in turn can affect offspring growth, behavior, and survival (‘maternal effects’; Bernardo 1996; Mousseau and Fox 1998; Krist 2011). In birds, variation in egg size has been shown to correlate with offspring quality (Williams 1994; Krist 2011), but eggs laid by the same female are often highly consistent in size across breeding attempts, indicating that females’ ability to adjust egg size may be limited (Christians 2002; Fortuna et al. 2021). Alternatively, egg components, such as nutrients and hormones, appear to vary to a greater extent with females’ prenatal environment (Groothuis et al. 2005; Eeva et al. 2011) and may thus be important alternative pathways for flexible maternal allocation (Saino et al. 2002; Williamson et al. 2006).

In cooperative breeders, females experience variable social conditions due to variation in the number of ‘helpers’ that assist with offspring care. Helpers provide food and other types of care to the offspring (e.g., protection from predators), often resulting in a positive correlation between number of helpers and offspring success and/or parental survival (Brouwer et al. 2005; Downing et al. 2020, 2021; D’Amelio et al. 2021), although sometimes varying in strength and direction depending on environmental conditions (Rubenstein 2011; Capilla-Lasheras et al. 2021a; Groenewoud and Clutton‐Brock 2021). Mothers could benefit from helpers’ presence by adopting one of two opposite prenatal reproductive strategies: (1) load-lightening, whereby females save energy by investing less in eggs when breeding with helpers, who compensate for this by providing food to the offspring, ultimately benefiting mothers’ survival and/or future reproduction (Russell et al. 2007; Taborsky et al. 2007), or (2) increased pre-birth investment when breeding with helpers, often called a differential allocation strategy (Russell and Lummaa 2009; Dixit et al. 2017), thereby increasing current offspring survival by investing more energy in reproduction when breeding under favorable conditions (i.e., life-history theory; Stearns 1992; Cunningham and Russell 2000; Sheldon 2000; Russell and Lummaa 2009; Savage et al. 2015; Valencia et al. 2017; Capilla-Lasheras et al. 2021b). Prenatal ‘load-lightening’ and ‘differential allocation’ have been mostly investigated for egg size, with no overall consensus (meta-analysis from Dixit et al. 2017 updated in Fortuna et al. 2021). To date, only two studies investigated whether maternal allocation in egg components may vary with helper presence (Russell et al. 2007; Paquet et al. 2013).

Egg components are crucial for offspring development, with the major source of nutrients and energy being yolk lipids and proteins (Carey 1996). Moreover, yolk carotenoids and vitamins influence the development of the embryo’s antioxidant and immune systems (reviewed in Biard et al. 2009), and enhance antioxidant responses and immunity in adulthood (Olson and Owens 1998; Surai et al. 2001). Nutrient-rich eggs result in better-quality offspring (Saino et al. 2003; McGraw et al. 2005; Biard et al. 2007), but nutrients are limited for females in natural environments, leading to a trade-off between the resources allocated to current offspring and the ones retained for the female (Erikstad et al. 1998; Blount et al. 2004). One study in superb fairy-wrens *Malurus cyaneus* analyzed 17 clutches and found evidence for load-lightening in yolk mass, lipids, and proteins in the presence of helpers (Russell et al. 2007). Instead, in sociable weavers *Philetairus socius*, no support was found for helper effects on egg carotenoid levels (of 84 clutches; Paquet et al. 2013).

In addition to nutrients, egg hormones may also be influenced by mothers’ social environment. In non-cooperatively breeding species, social factors have been shown to influence circulating androgens and corticosterone levels, and the concentration of these hormones in females’ eggs (Gil et al. 2007; Dentressangle et al. 2008; Safran et al. 2010; van Dijk et al. 2013; Bentz et al. 2016). Androgens, and particularly testosterone and androstenedione (A4) which have been extensively studied in birds, may enhance offspring competitive abilities, through faster development (Schwabl 1993; Eising et al. 2001) and stronger begging behavior (e.g., Eising and Groothuis 2003), but high levels of these hormones can also have harmful effects on offspring immune responses and survival, showing overall great variation in effect size and direction within and across the species studied (see reviews Groothuis et al. 2005; von Engelhardt and Groothuis 2011). Corticosterone, the primary glucocorticoid in birds, can be transferred from mothers to their eggs and has been shown to negatively influence offspring growth rate and body mass when present at high levels (Hayward and Wingfield 2004; Rubolini et al. 2005; Saino et al. 2005), but mother–egg transfers of corticosterone have similarly been suggested to program offspring to better survive in harsher environments (Hayward and Wingfield 2004; Love et al. 2013). In cooperative breeders, the only study that explored how helpers’ presence influences egg hormonal concentrations (Paquet et al. 2013) found that females without helpers laid eggs with more testosterone and A4, possibly to produce more competitive offspring (see also Cariello et al. 2006 for an example in joint-nest species). Further studies are thus needed to understand hormonal maternal allocation in cooperative breeders (Russell and Lummaa 2009; Bebbington and Groothuis 2021).

**Fig. 1:**
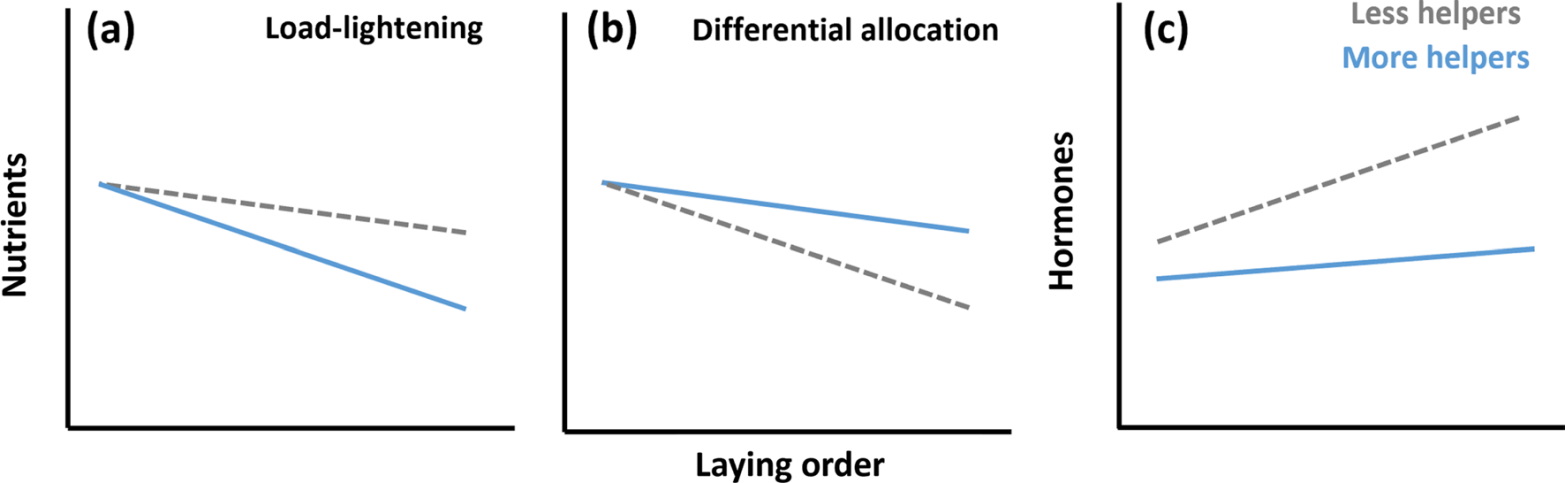
Predictions for the effect of helper number (less helpers than average represented by grey dashed line, more helpers than average by blue solid line) on how nutrients (**a** and **b**) and hormones (**c**) vary with laying order. In (**a**), the prediction is for ‘load-lightening’, whereby females with more helpers reduce nutrient allocation to eggs less likely to survive (i.e., latter-laid eggs). In (**b**), the prediction is for ‘differential allocation’, whereby females with more helpers allocate more resources toward later-laid eggs than females with less helpers. In (**a**) and (**b**), differences in nutrient allocation between females with more and less helpers should be more pronounced for latter-laid eggs and, even though different intercepts could also be expected, the previous results suggest no differences for first-laid eggs in this species (see Paquet et al. 2013). In (**c**), the prediction is for hormone variation, and is the same for the ‘load-lightening’ and the ‘differential allocation’ scenarios. Females with more helpers are expected to lay eggs with lower hormone levels (see Paquet et al. 2013). However, later-laid eggs are expected to have higher hormonal concentration than first eggs to mitigate hatching asynchrony effects on offspring survival, which should be stronger without helpers

Finally, an overlooked issue is whether helpers’ presence affects how mothers distribute resources within clutches. Eggs’ fitness value can vary with laying order and latter-laid eggs commonly have lower survival chances (Nager et al. 2000; Acevedo et al. 2020). In addition, variability in egg size and contents across the laying sequence has been well demonstrated (Slagsvold et al. 1984; Schwabl 1993; Kozlowski and Ricklefs 2010). ‘Cheaper’ components like hormones are often found to increase with egg laying order, which may increase the survival of later-hatched offspring (Royle 2001; Groothuis et al. 2005; Kozlowski and Ricklefs 2010), and costly components such as nutrients (Ojanen 1983; Williams 2005) often decrease across the laying sequence (Royle et al. 1999; Saino et al. 2002; Kozlowski and Ricklefs 2010). This reduction may be a consequence of nutrient depletion in female reserves and/or a strategy to allocate less resources to offspring that are less likely to survive (Slagsvold et al. 1984; Williams et al. 1993; Crean and Marshall 2009; Vedder et al. 2017). Since helpers may generally increase offspring survival (although such effect may be modulated by climatic conditions; Rubenstein 2011; Downing et al. 2020; Capilla-Lasheras et al. 2021a, b; D’Amelio et al. 2021; Groenewoud and Clutton‐Brock 2021), the adaptive value of laying later-laid eggs richer in hormones or poorer in nutrients could be modulated by helpers’ presence (Fig. 1), and it is therefore important to study how laying order and helpers’ presence interact to shape egg composition and offspring survival.

Here, we test whether maternal allocation to egg mass, yolk mass, yolk nutrients, and hormones varies with females’ breeding group size and laying order in the cooperatively breeding sociable weaver. First, we use data collected over seven breeding seasons to test whether and how offspring survival, via fledging probability, varies with laying order, which allows us to make predictions on how group size could modulate laying order effects. In sociable weavers, there is a size hierarchy among chicks due to hatching asynchrony. We then examine how egg mass and content vary with laying order and group size. We collected egg mass data during eight breeding seasons and, for egg content, we collected 59 clutches in two breeding seasons and measured nine egg components, representing three groups of compounds: yolk mass, lipids and proteins (macro-nutrients group), carotenoids, vitamin A and vitamin E (micro-nutrients group) and testosterone, A4, and corticosterone (hormones group). For nutrient allocation, we had opposite predictions according to the ‘load-lightening’ or the ‘differential allocation’ hypotheses (see Fig. 1). Finally, as a post hoc test given our results, we examine whether group size interacts with laying order effects on offspring hatching and fledging success.

## Methods

### Study system and data collection

Sociable weavers are a cooperatively breeding passerine endemic to southern Africa. They build communal nests, ‘colonies’, and each colony has several independent chambers where breeding pairs and helpers roost and breed (Maclean 1973a). Breeders can be assisted by one or several helpers with nestling feeding (Maclean 1973b), nest building and sanitation (Ferreira 2015). Helper number appears predictable by females at laying, as most helpers are previous offspring of the breeders (Covas et al. 2006; Fortuna et al. 2022), roosting group sizes before breeding were found to correlate with breeding group sizes (Paquet et al. 2016), and social bonds estimated via feeding associations, although present between birds from the same colony, are stronger within breeding groups (Ferreira et al. 2020).

Sociable weavers breed for several months (Mares et al. 2017) and can have up to 11 breeding attempts per season (Maclean 1973c; Fortuna et al. 2021). Clutch size typically ranges between 2 and 4 eggs and females lay one egg per day (Covas and Du Plessis 2005; Fortuna et al. 2021). The incubation period lasts around 15 days and nestlings normally hatch asynchronously (Maclean 1973c; Covas and Du Plessis 2005). The subsequent nestling period lasts for 21–25 days (Maclean 1973c).

This work was conducted at Benfontein Nature Reserve, Northern Cape Province, South Africa (28°520 S, 24°500 E), under permission from landowners, provincial authorities, and the UCT Ethics committee.

We monitored the breeding activity of 16 sociable weaver colonies during 8 breeding seasons (from 2010/2011 to 2017/2018) to obtain data on egg mass, egg laying order, and fledging success (see online Appendix A1 for details on data collection; D’Amelio et al. 2021; Fortuna et al. 2021). We obtained a sample of 779 eggs (in 326 nests from 14 colonies) with known mass and laying order, and for which breeding female’s identity, tarsus size, and group size were identified (see below). The sample of hatched eggs with known chick fate (fledged or not; see below), known egg mass and laying order, and known mother identity was of 419 (for 258 nests from 16 colonies; see below).

### Egg content

Over two non-consecutive breeding seasons, 2014 and 2017, we collected a total of 174 eggs (59 clutches) for content assessment. In 2014, 129 eggs (43 clutches) were collected between September and October, and in 2017, 45 eggs (16 clutches) were collected between October and December. This represented 7.5% of the clutches laid by this population in the two breeding seasons (*N* = 784 clutches) and is considerably lower than the estimated annual brood failure only due to predation (ca. 22.5%; see D’Amelio et al. 2021), to which sociable weavers usually respond by laying one or several replacement clutches (Covas et al. 2008; Fortuna et al. 2021). Sociable weavers can re-lay over ten times in the same season, and we therefore consider that egg removal had similar, or smaller, effects in their reproduction to egg loss under natural predation conditions (Covas et al. 2008; Fortuna et al. 2021). Furthermore, clutches laid by the same female were not collected twice (even across seasons).

Eggs were collected after weighing, 2 days after the first egg was found (most clutches have 3 eggs; 4th eggs were collected if found on the following day; Fortuna et al. 2021), and were stored whole by freezing at − 20 °C. From the 174 eggs collected, four eggs got damaged during transportation and only 170 could be analyzed.

We measured 9 egg components, representing 3 groups of compounds: yolk mass, lipids and proteins (macro-nutrients), carotenoids, vitamin A and vitamin E (micro-nutrients) and testosterone, A4, and corticosterone (hormones). Each batch of samples was analyzed during the season of collection, except corticosterone concentration of the 2014 samples which was measured at the same time as the 2017 samples. Hormonal assays for all eggs were conducted in the same laboratory (see Fanson et al. 2017).

Detailed methods of yolk contents’ analyzes are available in online Appendix A2–6. Briefly, yolks were separated from the albumen while defrosting and weighed at the nearest 0.001 g (online Appendix A2). Yolk lipids’ concentration was obtained by extraction with chloroform (online Appendix A3) and proteins’ concentration by CHN (determination of carbon, nitrogen, and hydrogen contents; online Appendix A4). Fresh yolk carotenoid concentrations were determined by colorimetry in 2014 and, in 2017, carotenoid concentration and composition were determined by reverse-phase high-performance liquid chromatography (HPLC; online Appendix A5; see Table S1 for description of carotenoid composition). Vitamin A (retinol) and vitamin E (sum of δ-, γ-, and α-tocopherol) concentrations were determined by HPLC (online Appendix A5; see Table S1). Yolk concentrations of testosterone, A4, and corticosterone were determined by radioimmunoassay (RIA) and enzyme-linked immunosorbent assay (ELISA; online Appendix A6). Sample sizes for each egg component can be found in Table S2. Correlations between egg components are given in Fig. S1.

### Group size and females’ identification

Individuals visiting the nests were identified using direct observations from 2010 to 2013/14, and by video recording nests for a minimum of 2 h from 2014/15 on (see Silva et al. 2018).

When possible, nests were observed/recorded more than once during the nestling period, as different individuals may visit the nests in different days and additional helpers can appear when nestlings are older (Ferreira A., personal communication). Since only some broods survive until fledging, and consequently, we do not have an accurate measure of maximum group size for all nests, group size was calculated as the mean number of birds seen visiting the nests over all observations of each breeding attempt. Only birds that appeared at least 3 times (in the same day or different days) were considered, to avoid including prospecting individuals that do not share the workload with the parents. Nest building visits were excluded and unringed birds were included (counted as 1 bird).

To identify breeding females, we used a combination of criteria: incubation video recordings (for collected clutches, recorded before collection) and video recordings or direct observations of feeding visits in current and/or posterior breeding attempts in the same nest and colony. We then used information from genetic analysis from blood samples (Paquet et al. 2015) and field data (Silva et al. 2018) to attribute parentage to the birds seen (see online Appendix A7 for details; Fortuna et al. 2021).

For the collected clutches, we identified the breeding female of 51 out of 59 clutches. The group size of these females could not be estimated during rearing due to collecting the eggs and was instead estimated from their subsequent breeding attempts for 46 out of 51 females. We expected that group size would not severely change in their next breeding attempt, as no juveniles were produced (since all eggs were collected) and most replacement clutches were laid within 2 months. Furthermore, we found a correlation of 0.57 (95 CI [0.33; 0.75]; *p* < 0.001; *N* = 50) between the size of two consecutive groups of the same breeding female, when using the long-term database (see details in online Appendix A8).

### Statistical analyses

Data were analyzed in R version 4.0.4 (R Development Core Team 2021).

#### Fledging probability

Before running egg mass and content models, we tested how fledging probability varied with laying order. This allowed us to make predictions on how helper number could modulate laying order effects (Fig. 1). For this, we fitted a binomial generalized linear mixed model (GLMM) with ‘fledged’ as a binary response variable (0 if the chick did not fledge, 1 if it did) and laying order as a continuous variable (as we predicted a linear increase/decrease in contents with laying order, see Fig. 1), while controlling for clutch size and egg mass as fixed effects and nest identity (i.e.: brood identity) and breeding female identity as random effects (see further details in online Appendix A9).

#### Egg mass

To test whether group size interacted with laying order, we fitted a linear mixed model (LMM) with egg mass as response variable and laying order, group size, and their interaction as variables of interest. As covariates, we included clutch size and mother tarsus size, which was previously found to explain egg mass variation (Fortuna et al. 2021) In the previous studies, short-term effects of rainfall and temperature on sociable weavers’ egg size and composition were not detected (Paquet et al. 2013; Fortuna et al. 2021) and we thus did not include climatic variables in our models, but accounted for inter-annual variation in climate by including a ‘season’ variable. The single effect of group size and covariates on egg mass will not be discussed here as a previous analysis was performed in an extension of the dataset used here (i.e., not including laying order, *N* = 1928; here *N* = 779; see Fortuna et al. 2021). The random terms’ structure included nest, breeding female, colony identity, and season. Spearman rank correlation coefficients were never above 0.26.

This and all models described hereafter were run in a Bayesian framework using the MCMCglmm package (Hadfield 2010), because it better accommodated random terms with low estimated variance (see Tables S4-S15 in online Appendix B). We scaled and centered all numerical independent variables to improve interpretation and comparison of effects and to enable interpreting main effects when these are included in an interaction (Schielzeth 2010), respectively. This was done by subtracting their mean and dividing by one standard deviation, and numerical response variables were scaled by dividing by one standard deviation. We used vague priors for all parameters (for details on model procedures, priors and diagnostics, see online Appendix A9). For each estimate, we present mean and 95% credible intervals of the posterior samples (or highest posterior densities intervals; 95CrI). We report effects as statistically credible when 95CrI do not overlap zero and discuss effects in which 95CrI slightly overlap zero.

#### Yolk mass and contents

To test if group size and laying order had interactive effects on egg content, we fitted separate LMMs using each component as response variable: yolk mass, carotenoids’ concentration, lipids percentage, proteins percentage, concentrations of vitamin A, vitamin E, testosterone, A4, and corticosterone (see online Appendix A9 for models’ details). Two random terms representing colony and mother identity were included (only one clutch was collected per breeding female across the two seasons), and fixed covariates: clutch size, season (due to only having 2 levels; to account for seasonal climatic effects and variation in laboratory procedures in the two seasons—see “Methods”), and predator-protection status as a binary factor, since some eggs in 2014 were collected in colonies where a predator-exclusion experiment was running (0 for control colonies, 1 for protected colonies; see Fortuna et al. 2021 for information on the experiment). We did not expect interactive effects of group size and laying order to differ between predation treatments and therefore did not consider a three-way interaction. For the yolk components measured in a smaller sample of eggs (vitamin A, vitamin E, and A4 concentrations; 36 eggs from 14 clutches; see Table S2), fixed covariates were not added to the model to avoid overparameterization and results should thus be interpreted with caution (none of these clutches was collected in predator-protected colonies). Since eggs/yolks of different weight could still be similarly rich in some nutrients/hormones (e.g., yolk mass decreases with laying order—see Results—but absolute quantity of carotenoids allocated is similar for all eggs), models were run with and without egg mass and yolk mass as covariates to estimate relative and absolute changes in content (see detailed explanation in online Appendix A9), but results in absolute terms are only mentioned below when they differed from relative changes.

#### Helper effects on offspring survival: post hoc test

Based on the egg content findings showing that females with more helpers laid eggs richer in nutrients (see Results), we predicted that these eggs could have a higher survival probability than eggs laid by females with fewer helpers, especially later-laid ones (see below). Therefore, as a post hoc analysis, we ran two models testing if group size and laying order had interactive effects on hatching and on fledging probability as binary response variables (0 if the chick did not hatch/fledge, 1 if it did). Models’ structure was the same as in the fledging probability model (see online Appendix A9), only adding group size in interaction with laying order. These binomial generalized linear mixed models (logit link) were fitted in MCMCglmm with priors for fixed and random terms as described above but fixing the prior residuals’ variance to 1 (Hadfield 2014). Latent variables were truncated to prevent under/overflow (Hadfield 2010). Number of iterations, burn-in, and thinning intervals were adjusted to ensure minimum effective sample sizes of 1000 (see code for details). Plots show raw data and the predicted effects estimated using the ‘predict’ function in MCMCglmm (Hadfield 2010). We present means [and 95CrIs] from the posterior distributions of interest in the results.

## Results

### Fledging probability

**Fig. 2:**
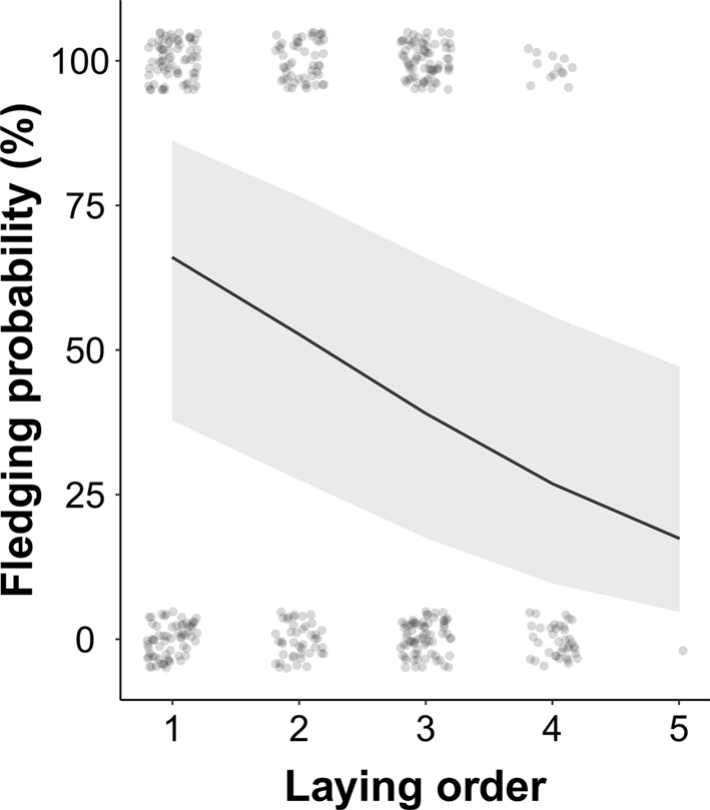
Raw data (*N* = 419) and predicted slope (with 95CI) of the relationship between fledging probability and laying order

Results showed that the probability of fledging was negatively correlated with the egg position in the laying sequence (Fig. 2; Table S3), suggesting that offspring reproductive value varies with laying order. We therefore predicted that helper number effects on maternal allocation would be more evident in later-laid eggs, i.e., offspring with lower survival probability (see Fig. 1).

### Egg mass

We found no evidence for an interactive effect of group size and laying order on egg mass (− 0.01 [− 0.05; 0.04]; *p* = 0.602; *N* = 779; Fig. S2; Table S4), but later-laid eggs were heavier than first-laid eggs (0.19 [0.14; 0.23]; *p* = 0.001; Fig. S2; Table S4).

### Egg components

#### Macro-nutrients: yolk mass, lipids, and proteins

**Fig. 3:**
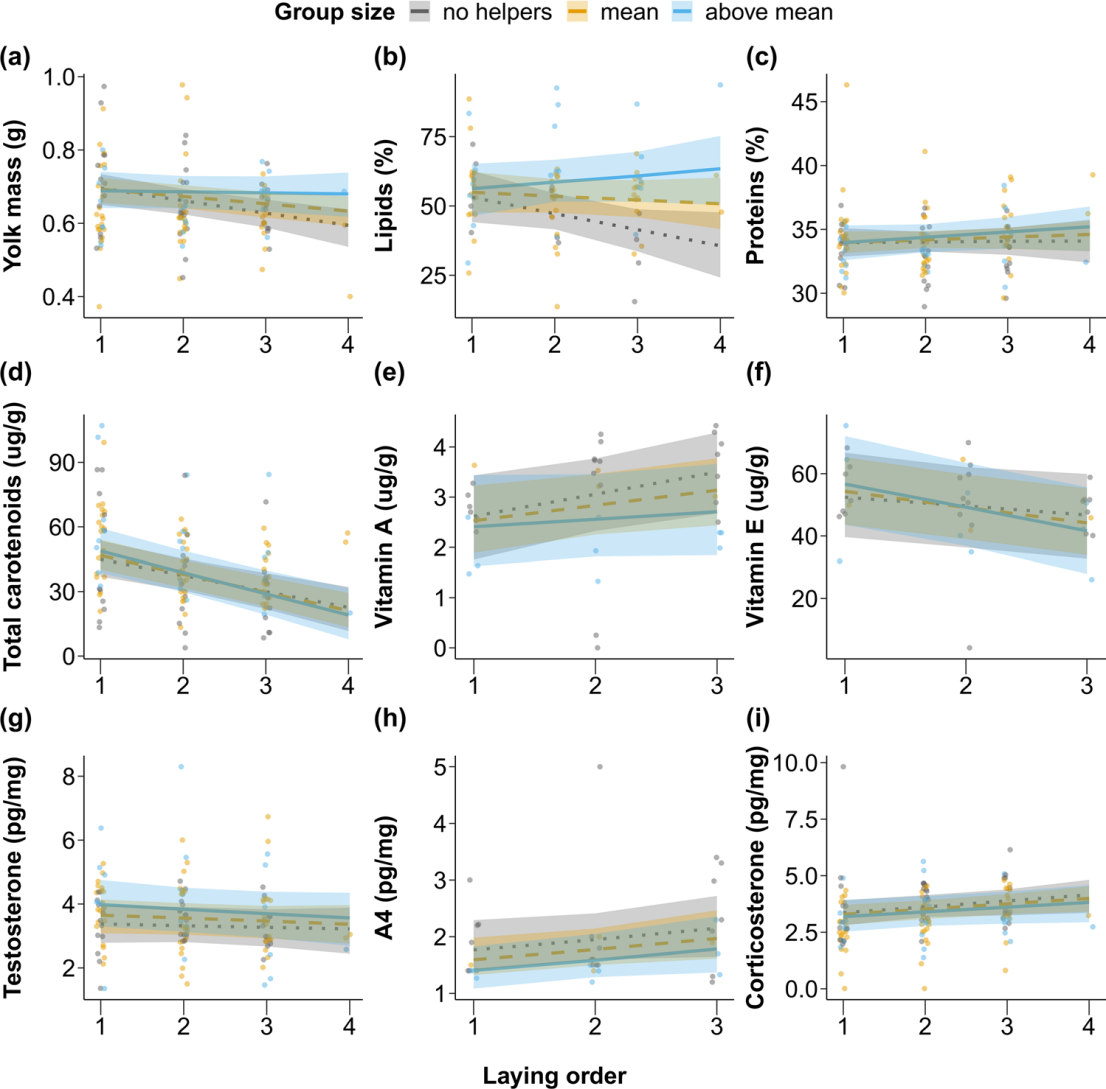
Relationship between egg components (**a** to **i**) and laying order for females with different group sizes. Lines represent the posterior predicted means and 95% credible intervals for three group size values: group size = 2 (no helpers; grey dotted line), mean group size (between 2.6 and 4 depending on dataset; orange dashed line) and the average between mean and maximum group size (between 3.3 and 5.6; blue solid line). Points represent raw data and point colors represent observations for groups without helpers, groups between group size = 2 and mean group size or group sizes above the mean (rounded to the nearest integer)

Yolk mass varied differently with laying order depending on group size (interaction = 0.12 [0.02; 0.24]; *p* = 0.034; *N* = 122; Figs. 3a and S3; Table S5). For females without helpers, fourth eggs’ yolk was predicted to be on average 0.1 g lighter than first eggs’ yolk (4th egg = 0.59 [0.54; 0.64]g; 1st egg = 0.70 [0.65; 0.73]g), representing a decrease of approximately 16%, while for females with a group size above average (approximately 4 helpers), this represented only a 1% decrease on average (4th egg = 0.68 [0.62;0.74]g; 1st egg = 0.69 [0.64; 0.74]g; Fig. 3a). There was no evidence for an overall effect of group size on yolk mass (0.11 [− 0.07; 0.30]; *p* = 0.276; Figs. 3a and S3; Table S5). Yolk mass, in terms of proportion of yolk in relation to egg mass, varied negatively with laying order (− 0.16 [− 0.27;-0.04]; *p* = 0.004; Figs. 3a and S3; Table S5), while absolute changes in yolk mass followed a similar trend but not as clear statistically (Table S5).

Yolk lipids’ concentration was higher when females had larger group sizes, especially in later-laid eggs (interaction = 0.21 [0.01; 0.39]; *p* = 0.030; *N* = 83; Fig. 3b; Table S6). Females without helpers were predicted to lay fourth eggs with 17% less yolk lipids than first eggs (4th egg = 36 [24; 48]%; 1st egg = 53 [44; 63]%), whereas females with group sizes above average laid fourth eggs with on average 7% more yolk lipids than the first egg (4th egg = 63 [50; 75]%; 1st egg = 56 [46; 65]%; Fig. 3b). In general, females with more helpers produced eggs richer in yolk lipids (0.34 [0.08; 0.55]; *p* = 0.012; Figs. 3b and S3; Table S6).

For yolk proteins, we found no evidence for effects of the interaction (0.04 [− 0.13; 0.21]; *p* = 0.652; *N* = 117; Figs. 3c and S3; Table S7) or for group size (0.07 [− 0.16; 0.32]; *p* = 0.580; Figs. 3c and S3; Table S7) and laying order (0.07 [− 0.11; 0.24]; *p* = 0.462; Figs. 3c and S3; Table S7) as single terms.

#### Micro-nutrients: carotenoids, vitamin A, and vitamin E

We found no evidence that group size interacted with laying order to explain variation in carotenoids (− 0.05 [− 0.14; 0.06]; *p* = 0.350; *N* = 119; Figs. 3d and S3; Table S8), vitamin A (− 0.13 [− 0.37;0.12]; *N* = 36; *p* = 0.264; Figs. 3e and S3; Table S9), and vitamin E concentrations (− 0.16 [− 0.33; 0.04]; *p* = 0.088; *N* = 36; Figs. 3f and S3; Table S10).

There was also no evidence for a main effect of group size on yolk carotenoid (0.03 [− 0.22; 0.28]; *p* = 0.812; Figs. 3d and S3; Table S8), vitamin A (− 0.27 [− 0.78; 0.29]; *p* = 0.256; Figs. 3e and S3; Table S9), and vitamin E concentrations (0 [− 0.79; 0.64]; *p* = 0.974; Figs. 3f and S3; Table S10).

Laying order correlated negatively with yolk carotenoid (− 0.35 [− 0.46; − 0.25]; *p* = 0.001 Figs. 3d and S3; Table S8) and vitamin E concentrations (− 0.30 [− 0.48; − 0.10]; *p* = 0.006; Figs. 3f and S3; Table S10) and tended to correlate positively with vitamin A (0.23 [− 0.02; 0.48]; *p* = 0.070; Figs. 3e and S3; Table S9).

#### Hormones: testosterone, A4, and corticosterone

We found no support for an interactive effect of group size and laying order on hormonal concentration, namely on testosterone (− 0.03 [− 0.16; 0.08]; *p* = 0.624; *N* = 122; Figs. 3g and S3; Table S11), A4 (0.03 [− 0.21; 0.28]; *p* = 0.838; *N* = 36; Figs. 3h and S3; Table S12), and corticosterone (− 0.02 [− 0.13; 0.11]; *p* = 0.758; *N* = 122; Figs. 3i and S3; Table S13).

Contrary to expected, there were no detectable main effects of group size on testosterone (0.21 [− 0.09; 0.50]; *p* = 0.146; Figs. 3g and S3; Table S11), A4 (− 0.36 [− 0.78; 0.11]; *p* = 0.098; Figs. 3h and S3; Table S12) or corticosterone (− 0.08 [− 0.34; 0.22]; *p* = 0.584; Figs. 3i and S3; Table S13).

Finally, we found no support for laying order effects on testosterone (− 0.07 [− 0.20; 0.05]; *p* = 0.262; Figs. 3g and S3; Table S11). However, later-laid eggs had higher corticosterone concentration (0.16 [0.01; 0.27]; *p* = 0.014; Figs. 3i and S3; Table S13) and tended to have higher A4 concentration (0.25 [0; 0.50]; *p* = 0.056; Figs. 3h and S3; Table S12).

### Interaction between laying order and group size on offspring survival

**Fig. 4:**
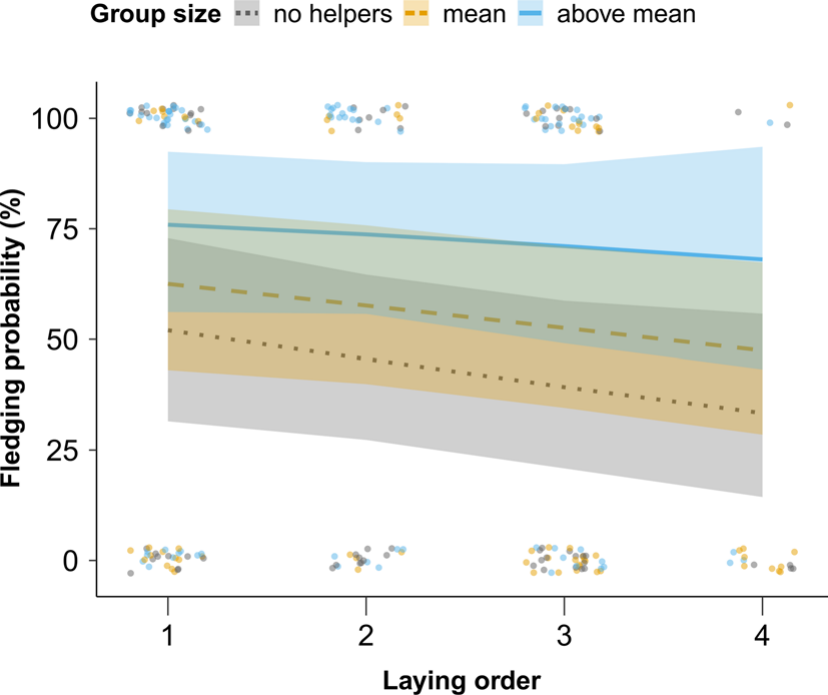
Relationship between fledging probability and laying order for females of different group sizes. Lines represent the posterior predicted means and 95% credible intervals for three group size values: group size = 2 (no helpers; grey dotted line), mean group size (3.3; orange dashed line), and the average between mean and maximum group size (5.2; blue solid line). Points represent raw data and point colors represent observations for groups without helpers, groups between group size = 2 and mean group size or group sizes above the mean (all values rounded to the nearest integer)

Post hoc analyses showed no evidence for an interaction between laying order and group size on hatching (OR 0.92 [0.66; 1.25]; *p* = 0.620; *N* = 331; Fig. S4; Table S14) or fledging probabilities (OR 1.20 [0.56; 2.52]; *p* = 0.649; *N* = 226; Fig. 4; Table S15).

At hatching, there were no detectable group size main effects (OR 1.02 [0.75; 1.43]; *p* = 0.880; Fig. S4; Table S14), but later-laid eggs were less likely to hatch than earlier-laid ones (OR 0.48 [0.34; 0.66]; *p* < 0.001; Fig. S4; Table S14).

Fledging probability correlated positively with group size as a single term (OR 4.77 [1.51; 16.26]; *p* = 0.002; Fig. 4; Table S15). Females without helpers were predicted to lay eggs with a fledging probability of 44% [25; 63], while eggs from females with an average group size were estimated to survive until fledging 57% [39;75] of the times, and 73% [0.54; 0.90] of the times when laid by females with a group size above average (Fig. 4). In accordance with the first fledging probability model (larger dataset than here, see Fig. 2 and online Appendix B1), later-laid eggs tended to have lower fledging probabilities (OR 0.53 [0.28; 1.00]; *p* = 0.047; Fig. 4; Table S15).

## Discussion

Here, we tested whether maternal allocation to egg mass, yolk nutrients—yolk mass, lipids, proteins, carotenoids, vitamins A and E—and hormones—testosterone, A4, and corticosterone—varied with group size and laying order in sociable weavers. Our aim was to investigate if females adopt flexible prenatal reproductive strategies as their number of helpers varies that could increase offspring’s and/or their own fitness. We first showed that laying order was negatively associated with fledging success, implying that eggs’ reproductive value varies within clutches. We then obtained two results concurring with the ‘differential allocation’ predictions, as later-laid eggs of females with more helpers had heavier yolks and more lipids when compared to females with fewer helpers and, overall, females with more helpers laid eggs richer in lipids. In contrast, remaining yolk nutrients and hormones were independent of females’ group size. As expected, latter-laid eggs had lower antioxidant levels but were more concentrated in corticosterone and A4. Finally, we ran a post hoc test examining if eggs laid by females with more helpers, especially later-laid ones, would show higher survival, which could be partially explained by the detected differences in yolk mass and lipids. This analysis showed that females with more helpers laid eggs with a higher fledging, but not hatching, probability, and that this was independent of laying order. This suggests that ‘differential allocation’ of some egg nutrients when females breed with more helpers could be improving offspring fitness overall but may not translate into a higher survival specific to later-laid eggs.

### Helper effects on egg allocation

In this population, nests with more helpers were seen to receive more food (Covas et al. 2008) and suffer less brood reduction (D’Amelio et al. 2021). Furthermore, here, we found that fledging probability was negatively correlated with eggs’ laying order. Altogether, this implies that offspring survival varies with helper number and laying order, and that helper effects may interact with laying order effects on offspring survival. We therefore expected flexible maternal allocation strategies in relation to group size and laying order.

When testing the effects of this interaction on maternal allocation to eggs, we found that yolk mass and lipids’ concentration varied with laying order in different ways depending on females’ group size. Later-laid eggs of females with more helpers had heavier yolks and more lipids when compared to females with fewer helpers. These results concur with the predictions for a ‘differential allocation’ strategy in relation to helpers’ presence and laying order (Fig. 1). The adaptive value of intra-clutch variation in egg investment has long been proposed, with some species suggested to follow a ‘brood-reduction strategy’ and others a ‘brood-survival strategy’ (Slagsvold et al. 1984). Based on our results, sociable weaver females might swing between these two strategies depending on their number of helpers (Russell and Lummaa 2009), via flexible allocation of yolk and lipids to their eggs. Offspring that develop from eggs with heavier yolks and more lipids should have access to more energy and nutrients, which are vital for embryonic tissue growth, and chicks should hatch with greater nutrient reserves that can be used for several days post-hatching (Noble and Cocchi 1990; Williams 1994; Carey 1996). Furthermore, some lipid constituents, namely fatty acids, have been reported to correlate with offspring hatching and fledging success (Mentesana et al. 2021). Therefore, in cooperative breeders, this ‘differential allocation’ strategy could be adaptive if a higher maternal investment in egg nutrients summed with the extra food provided by the helpers increases the survival probability of offspring from later-laid eggs, thus increasing the number of offspring reaching independence (D’Amelio et al. 2021). In contrast, when breeding with less helpers, females could benefit from a biased allocation of nutrients toward eggs with higher reproductive value (i.e., earlier-laid eggs), saving energy for their own survival or future reproduction if brood reduction is likely to occur through later-hatched chicks’ mortality (Williams et al. 1993; Royle et al. 1999; Crean and Marshall 2009; Vedder et al. 2017). It should be noted though that the differences observed here for yolk mass do not appear to result from variation in yolk lipids or proteins, as these variables were not clearly correlated with yolk mass, and may instead represent an increase in other minor dry components, as minerals and carbohydrates, or water content (Nys and Guyot 2011).

Besides, we found that females with more helpers laid eggs richer in lipids independently of laying order, which implies that mothers may be allocating more nutrients to all eggs when breeding with more helpers. The idea that sociable weaver females invest more when breeding in better conditions concurs with the previous results in this species showing that females laid larger clutches in better climatic conditions and in colonies protected from nest predation (however, no change in egg mass or number was found in relation to helpers: Fortuna et al. 2021). ‘Differential allocation’ was first proposed as a beneficial strategy when females mate with attractive partners (Burley 1986; Cunningham and Russell 2000; Sheldon 2000), but was later suggested to explain cases in which females provided more care when breeding with more helpers (Russell and Lummaa 2009; Dixit et al. 2017). However, evidence that ‘differential allocation’ has evolved as a prenatal strategy in cooperative breeders is scarce and limited to egg size, having been reported only once in Iberian magpies *Cyanopica cooki* (Valencia et al. 2017; see also Woxvold and Magrath 2005; Lejeune et al. 2016 for reports of positive helper effects on clutch size). Moreover, results supporting the opposite strategy, ‘load-lightening’, via egg size and nutritional content are, respectively, ambiguous (Dixit et al. 2017; Fortuna et al. 2021) and rare (Russell et al. 2007). More studies are needed before concluding on the generality of ‘differential allocation’ through egg components across cooperative breeders. However, theoretical work by Savage et al. (2015) predicts that females should take advantage of better rearing conditions by increasing prenatal investment, if this investment leads to lasting benefits for offspring and allows them to receive more post-birth care. In sociable weavers, there is some evidence that prenatal investment may affect offspring begging behavior (Paquet et al. 2015), and thus, the rate at which nestlings are fed (Fortuna et al. 2022), suggesting that it may prime offspring to receive more postnatal care. Yet, ‘differential allocation’ is not expected when early investment is unimportant or interchangeable with postnatal investment (Savage et al. 2015), and it would therefore be relevant to assess the effects of maternal allocation to yolk mass and lipids on offspring quality and survival in this species (see also below).

Differences in egg nutrients could be explained by differences in female quality/condition, if better females can lay eggs, or later-laid eggs, with more nutrients (Ardia et al. 2006) and also have more helpers, or if having helpers in past breeding events improves females’ condition in the following reproductive attempt. The link between female quality/condition and helper number in sociable weavers is not clear. For example, not all females with more helpers seem to survive better, but only younger ones (Paquet et al. 2015). Even though we attempted to account for female quality and condition in our models, using proxies as clutch size and egg mass (Fortuna et al. 2021), we cannot determine whether the ‘differential allocation’ pattern found here is a consequence of females being in better state or an adjustment of egg content to helpers’ presence that is independent of females’ condition (Cockburn et al. 2008; Russell and Lummaa 2009).

An experimental manipulation of helper number in females’ groups could help to disentangle female quality/condition from helper effects (but see Cockburn 1998). Otherwise, this could be achieved with longitudinal studies that follow females as their group size varies (Fortuna et al. 2021). The latter could also provide valuable insights on how prenatal allocation strategies may be moderated by other conditions of the females’ environment, such as variation in climatic conditions prior to laying, which do not seem to explain variation in egg size in sociable weavers but may influence maternal allocation to egg composition (Hatchwell 1999; Langmore et al. 2016; Fortuna et al. 2021).

We found no evidence that egg mass and remaining yolk nutrients—proteins, carotenoids, vitamin A, and vitamin E—varied in relation to the group size alone or in interaction with laying order (see also Fortuna et al. 2021). Egg mass was positively correlated with laying order, as previously found is this population (van Dijk et al. 2013) and other species (Howe 1976; Zach 1982; Slagsvold et al. 1984; Rutkowska and Cichon 2005). Our results also show that, even though later-laid eggs were heavier, these had proportionally lighter yolks and lower carotenoids and vitamin E concentrations. Moreover, we did not detect relationships between egg mass and yolk lipids, proteins or hormones in the eggs, which suggests that studying egg mass may provide only partial insights on egg quality (see also Hadfield et al. 2013).

Surprisingly, we found no effect of group size on eggs’ hormonal content. This contradicts the previous findings in this species, where the first egg of the clutches was found to be more concentrated in testosterone and A4 for females without helpers (Paquet et al. 2013). Instead, our results indicate that females without helpers may not benefit from allocating more hormones to offspring, or specifically to chicks from later-laid eggs, that could enhance their competitive abilities. However, the contrasting results obtained here and before (Paquet et al. 2013) also suggest that other unaccounted environmental or social factors might affect egg hormonal levels. Discrepancies in hormonal effects are often detected possibly because maternal hormone transfers to eggs depend on several social and environmental cues (Groothuis et al. 2019; Bebbington and Groothuis 2021), which could also explain the inconsistencies found in this system. Nevertheless, the positive relationship between corticosterone concentration and laying order observed here, along with the tendency for A4 to positively correlate with laying order, concur with the literature reporting higher hormonal levels in later-laid eggs (Royle 2001; Kozlowski and Ricklefs 2010; Müller and Groothuis 2013). This could function as a ‘cheap’ mitigation strategy (Groothuis and Schwabl 2008) to enhance the competitive abilities of chicks from later-laid eggs, which hatch later and have less access to carotenoids and vitamin E (Royle 2001; this study).

### ‘Differential allocation’ and offspring survival

We then assessed if females breeding with more helpers had higher reproductive success, which could be partially mediated by laying eggs with heavier yolks and richer in lipids (i.e., ‘differential allocation’; Russell and Lummaa 2009). For this, we used the long-term dataset to test the interactive effect of group size and laying order on hatching and fledging success. Results showed no effect of this interaction on nestling survival, suggesting that chicks hatching from later-laid eggs that are raised with more helpers do not appear to have an advantage in terms of fledging success over the remaining chicks.

There are several possible explanations for the lack of detectable interactive effects between laying order and group size on hatching and fledging success. If differences in offspring survival across the laying sequence are solely explained by hatching asynchrony, i.e., independent of maternal allocation to eggs, this result suggests that having more helpers does not offset the disadvantage of later-hatched chicks. Yet, offspring survival differences with laying order could be influenced by the observed differences in egg composition, although here we cannot directly test how egg composition relates to offspring survival (because eggs are collected to analyze their components). If egg composition has an effect on offspring survival, not detecting a survival advantage of ‘differential allocation’ here may suggest that egg nutrients influence survival at other stages, for instance during the first days after hatching, or contribute instead to offspring morphological traits as body mass or size (Moore et al. 2019). Moreover, positive effects of increased allocation toward later-laid eggs may be undetectable at fledging if, for instance, they are masked by postnatal care. Another possibility is that environmental factors are mediating maternal allocation strategies in relation to group size (Langmore et al. 2016). Under this scenario, mothers distribute resources within clutches depending not only on helper number, but also on remaining environmental factors that differ between years (Langmore et al. 2016), resulting in undetectable general effects on offspring survival over the seven breeding seasons included in this analysis. Therefore, at this stage, we cannot fully dismiss that ‘differential allocation’ when breeding with more helpers has positive effects on offspring from later-laid eggs in this species, and further work focusing on offspring phenotypic traits and survival at different stages, and on seasonal variation in egg composition, would be necessary to assess these effects.

Nevertheless, our findings that group size is positively correlated both with eggs’ lipid content and with chicks’ fledging success suggest that ‘differential allocation’ may be contributing to higher offspring survival, independently of laying order. Therefore, females may be benefiting from the improved breeding conditions provided by helpers and increasing their reproductive output (Sheldon 2000; Russell and Lummaa 2009). Here, we estimated that females with three helpers were predicted to lay eggs with almost 30% more fledging chances than females without helpers. In accordance, previous analyses in sociable weavers showed positive helper effects on fledging mass and success under adverse conditions (Covas et al. 2008), and more recent long-term analyses showed that pairs with more helpers have a higher probability of fully-fledging their broods (D’Amelio et al. 2021). It would now be important to specifically address whether these benefits result from ‘additive’ effects of increased maternal allocation and helper care or solely from the postnatal contributions of helpers (Covas et al. 2008; Paquet et al. 2016).

## Conclusion

We have shown that maternal egg allocation in relation to helpers’ number may be detected for some egg components that are important for offspring development and survival. Females with more helpers laid eggs richer in lipids and their offspring had higher fledging success, which suggests that larger breeding groups represent improved breeding conditions for females. Moreover, females with more helpers produced later-laid eggs with heavier yolks and more lipids. This might imply that helpers’ presence modulates resource distribution within clutches. Future research should focus on the mechanisms leading to such ‘differential allocation’, to clarify whether this is a passive consequence of better female quality/condition or a strategy to take advantage of helpers’ presence. Second, studies across cooperatively breeding species, spanning a larger number of years and environmental conditions, are necessary to assess general patterns in maternal allocation to egg components. Finally, it is important to test if increases in yolk mass and lipids have fitness advantages for the offspring.

## Supplementary Information

Below is the link to the electronic supplementary material.

Supplementary file1 (DOCX 537 KB)

## Data Availability

Data and code to reproduce the analyses in this manuscript can be found at https://osf.io/raupk/?view_only=4283a309450948b88b3ba729f2362310.
