# Supplementary material for: Egg components and offspring survival vary with group size and laying order in a cooperative breeder
Source: Oecologia. 2023 May 6;202(1):129–42. doi: 10.1007/s00442-023-05379-w (PMC10229689; doi:10.1007/s00442-023-05379-w)
Supplement: Supplementary file 1 — Supplementary file1 (DOCX 537 KB) [file 442_2023_5379_MOESM1_ESM.docx]

# **Appendix of the manuscript: “Egg components and offspring survival vary with group size and laying order in a cooperative breeder”**

Rita Fortuna, Matthieu Paquet, Clotilde Biard, Loïc Élard, André C. Ferreira, Mathieu Leroux-Coyaux, Charline Parenteau, Liliana R. Silva, Franck Théron, Rita Covas and Claire Doutrelant

**Corresponding author**: Rita Fortuna (rita.fortuna@ntnu.no)

**Contents**

[Appendix 1](#_Toc96951047)

[**A.** **Methods** 2](#_Toc96951048)

[1. Captures and breeding monitoring 2](#_Toc96951049)

[2. Yolk mass 2](#_Toc96951050)

[3. Yolk lipids 3](#_Toc96951051)

[4. Yolk proteins 3](#_Toc96951052)

[5. Yolk carotenoids and vitamins 4](#_Toc96951053)

[6. Yolk testosterone, androstenedione (A4) and corticosterone 6](#_Toc96951054)

[7. Identification of breeding females 9](#_Toc96951055)

[8. Group size of females from collected clutches 10](#_Toc96951056)

[9. Statistical analyses 10](#_Toc96951057)

[Fledging probability model 10](#_Toc96951058)

[Egg mass, yolk mass and contents models 11](#_Toc96951059)

[**B.** **Results** 14](#_Toc96951060)

[1. Fledging probability 14](#_Toc96951061)

[2. Egg mass 15](#_Toc96951062)

[3. Egg contents 17](#_Toc96951063)

[Yolk mass, lipids and proteins 17](#_Toc96951064)

[Total carotenoids, vitamin A and vitamin E 20](#_Toc96951065)

[Testosterone, A4 and corticosterone 23](#_Toc96951066)

[4. Helper effects on hatching and fledging success 27](#_Toc96951067)

[**References** 29](#_Toc96951068)

## **Methods**

### Captures and breeding monitoring

Individuals were captured once or twice a year at the colonies using mist nests since 1999 (Covas 2002). Birds were ringed with a uniquely coded aluminum ring and a unique color-ring combination, allowing individual visual identification, and blood samples were collected for genetic sexing and determination of parentage relationships (see below).

Nest contents were inspected every 3 days from mid-September. First eggs were marked with a pencil and nests were inspected every day to mark every subsequently laid egg. Two days after clutch initiation, we weighed the eggs to the nearest 0.001 g with a digital Pesola scale. Nests were checked the following day to weigh a possible 4^th^ egg. We did not intentionally look for 5^th^ eggs because five eggs’ clutches are rare (4% of the clutches; Fortuna et al. 2021), but all nests were routinely inspected every 3 days and 5^th^ eggs were weighed whenever found.

Nests were monitored until hatching and, when possible, we marked chicks according to their egg of origin (if two chicks hatched in the same day, egg of origin was unknown). When the first nestling was 9 days old, nestlings were weighed and ringed with a unique numbered aluminum ring and a blood sample was taken. When the first chick was 17 days of age (day 17 of the nest; the last day nests can be visited without increasing the chances of inducing fledging), nestlings’ wing, tarsus, and weigh were measured. The fate and fate date of each egg and chick were registered and if the chicks survived until day 17 they were considered as having fledged.

### Yolk mass

The yolks were separated from the albumen while defrosting, weighed at the nearest 0.001g and kept at -80°C until analyses. Albumen mass (see Table S2) was estimated as the difference between egg mass (before freezing, weighed in the field at collection) and wet yolk mass, since wet egg mass could not be reliably estimated due to frequent albumen leaks through eggshell cracks caused by freezing and transportation.

### Yolk lipids

For lipids estimation, yolks were then dried at 60ºC for about 24-48 hours. We introduced 180 mg of dried yolk into tubes resistant to chloroform (type Sarstedt 15 mL). Then, 3 mL of deionized (DI) water, 6 mL of methanol and 3 mL of chloroform were added to the samples (Bligh and Dyer 1959). Afterwards, the samples were vortexed (30 seconds at 2400 rpm), 3 mL of DI water and 3 mL of chloroform were added and then vortexed again and centrifuged (10 minutes at 4500 rpm). Samples were biphasic, the water and methanol were positioned on the top, the chloroform and lipids on the bottom and one thin layer of proteins divided the two phases. The chloroform and lipids were extracted with a Pasteur pipette into a glass tube previously weighed and were reserved. Then, 3 mL of chloroform were added again to the sample, which was vortexed and centrifuged. We did a second extraction of the chloroform and added it to the previous extraction. The tubes with the extractions were put in heating plates (60ºC) and when all chloroform evaporated, tubes containing the lipids were weighed.

### Yolk proteins

Yolks were dried at 60ºC for about 24-48 hours. The nitrogen concentration of the samples was determined with an elemental analyzer Thermo-Finnigan, Flash EA 1112 Series. Protein content was calculated from the nitrogen values using a conversion factor of 6.25. About 2 mg of dried yolk was weighed in a microbalance (Sartorius MC5) in a tin capsule, sealed and placed in an auto sampler, from which it was dropped into a combustion chamber. As the sample entered, the combustion chamber oxygen was injected into the carrier gas (He), which flowed through the combustion tube. The temperature raised up to 1800°C, which insured complete combustion of the sample. Inter and intra variations were never above 5.10% and 2.34%, respectively.

### Yolk carotenoids and vitamins

Carotenoids’ concentration of the samples collected in 2014 was determined by colorimetry following procedures in Paquet et al. (2013). For carotenoid extraction, 60 mg of egg yolk was diluted with acetone (1μg of acetone for 0.1mg of yolk). Samples were vortexed, kept overnight at 20°C and then centrifugated (10 minutes at 13000g, at 4ºC). We extracted 125 μL of supernatant for each sample and determined the optic density (OD) at 450nm in a microplate photometer (Victor3 1420 Multilabel Plate Reader, Perkin-Elmer). Commercial solution of lutein (xanthophylls Sigma X-6250) was used for serial dilution and to obtain a standard curve to determine the relationship between the OD value and carotenoid concentration in yolk eggs, expressed as µg/g yolk. We used the mean of the two closest values obtained for the three replicates as the carotenoid concentration in fresh yolk eggs.

Carotenoid composition and concentration, as well as vitamin A and vitamin E concentrations, of the samples collected in 2017 were determined by reverse phase high performance liquid chromatography (HPLC), using a Jasco equipment (PU-2089 Quaternary Gradient HPLC pump, AS-2057 auto sampler, MD-2018 Diode-array Detector and FP-2020 fluorescence detector; monitored through ChromNAV software; Jasco France), following procedures adapted from Biard et al. (2005; 2009).

Lipid-soluble antioxidants were extracted from eggs using half the yolk (mean yolk mass used ± s. e. = 0.35 ± 0.06 g). Yolk samples were homogenized with 0.7 mL NaCl 5% and 1 mL ethanol with an IKA T10 Basic Ultra Turrax tool (Fisher Scientific). Antioxidants were extracted adding 2 mL hexane and further homogenization, centrifugation and collection of the hexane phase (extraction repeated twice for all samples and three times for nine samples when yolk was still visible in the tubes after the second extraction). Hexane extracts were pooled and evaporated in a block heater at 37°C under nitrogen flow. The residue was dissolved in 0.4 mL dichloromethane and 0.4mL methanol. Sample extracts were filtered through Whatman NYL 0.45 μm syringe filter (Sigma Aldrich). Carotenoid composition was determined by injecting 40µL of extract onto a VENUSIL AQ C18 5µm C18 reverse-phase column, 25 cm×4.6 mm (Agela). An isocratic HPLC at a flow rate of 2 mL min^-1^ with a mobile phase of acetonitrile-methanol (60:40) for 8 minutes was used with a change within 1 minutes to acetonitrile-methanol-dichloromethane (60:20:20) and running for the next 11 minutes, followed with re-equilibration with the first mobile phase for the next 5 minutes, using detection by absorbance at 445 nm. Peaks were identified and concentrations calculated by comparison with the retention times and dilution curves of a range of carotenoid standards (Sigma Aldrich). Total carotenoid concentration was also determined by injecting 40µL of extract onto a PROMOSIL 50µm NH2 reverse-phase column (25 cmx4.6 mm, Agela) with a mobile phase of methanol and HPLC grade distilled water (97 : 3), at a flow rate of 1.5 mL.min^-1^. Lutein was used for calibration (Sigma Aldrich). The correlation between total carotenoid concentration and the sum of the concentration of all peaks identified was of 0.95 (95CI=[0.91, 0.97]; P-value<0.001; N=41) and thus the sum of all identified peaks was used as measure of total carotenoid concentration. Total carotenoid concentration was highly positively correlated with the concentration of most of the identified carotenoid compounds (Table S1; Spearman rank’s correlation coefficient between 0.77-0.94, p<0.001, N=45), moderately positively correlated with β-Carotene concentration (correlation coefficient = 0.43, p=0.003) and not significantly correlated with Cryptoxanthin concentration (correlation coefficient = 0.05, p=0.763).

Concentrations of vitamins A (retinol) and vitamin E (δ-, γ- and α-tocopherol) were determined by injection of 40µL of extracts onto a UNISOL 3µm C18 reverse-phase column, 15 cm × 4.6 mm (Agela) with a mobile phase of methanol, at a flow rate of 1.5 mL min^-1^ using fluorescence detection by excitation and emission wavelength of 295 and 330 nm, respectively, for vitamin E and UV detection at 325 nm for vitamin A. Peaks were identified and concentrations calculated by comparison with the retention times and dilution curves of standards of retinol, δ-, γ- and α-tocopherol (Sigma Aldrich). All concentrations are expressed as µg/g yolk. Concentrations rather than quantities of antioxidants were used as dependent variables in subsequent analyses because concentration is the main factor determining physiological action of antioxidants at the level of tissues (Biard et al. 2009).

**Table S 1**. Composition of egg yolk in carotenoid and vitamin E compounds, as mean concentration (± SD), mean proportion (% ± SD) and proportion of samples (out of N=45) in which they were detected.

|  |  | Mean concentration ± SD | Mean % of total ± SD | Proportion of samples (%) |
| --- | --- | --- | --- | --- |
| **Individual carotenoids**  **(µg/g yolk)** | Lutein | 11.55 ± 5.61 | 54.9 ± 6.1 | 100 |
|  | Zeaxanthin | 2.29 ± 0.99 | 11.4 ± 2.3 | 100 |
|  | Cis-lutein | 1.81 ± 1.11 | 8.35 ± 2.61 | 96 |
|  | Cis-Zeaxanthin | 1.61 ± 0.66 | 8.19 ± 2.44 | 98 |
|  | β-Carotene | 0.51 ± 0.4 | 2.47 ± 1.71 | 89 |
|  | Cryptoxanthin | 1.34 ± 0.61 | 7.62 ± 5.04 | 96 |
|  | Unidentified carotenoids | 1.44 ± 0.63 | 7.07 ± 1.62 | 100 |
| **Vitamin E compounds (µg/g yolk)** | α-tocopherol | 33.3 ± 10.49 | 64.65 ± 11.26 | 100 |
|  | δ-tocopherol | 17.56 ± 7.79 | 33.52 ± 11.19 | 100 |
|  | γ-tocopherol | 1.01 ± 0.91 | 1.83 ± 1.43 | 98 |

### Yolk testosterone, androstenedione (A4) and corticosterone

The yolks were separated from the albumen while defrosting, weighed at the nearest 0.001g and kept at -20°C until analyses. Testosterone, androstenedione, and corticosterone of eggs collected in the two breeding seasons were assayed with RIA and ELISA methods (radioimmunoassay and enzyme linked immunosorbent assay, respectively).

In detail, 100 mg of each sample were homogenized in 1 mL of distilled water and three to four glass beads, using a vortex. Steroids were extracted by adding 3 mL of diethyl-ether to 300 μL of the mixture, vortexing and centrifuging (5 minutes at 2000 rpm, at 4°C). The diethyl-ether phase containing steroids was decanted and poured off after snap freezing the tube in an alcohol bath at minus 40°C. This was done twice for each yolk, and the solvent was then evaporated at 37°C. The dried extracts were re-dissolved in 800 μL of phosphate 0.01 M pH 7.4 buffer each hormone was assayed in duplicate. Then, for testosterone and corticosterone (in 2014 and 2017 samples) measured with RIA method, 100 μL of extract were incubated overnight at 4^o^C with 4000 cpm of the appropriate H^3^-steroid (Perkin Elmer, US) and polyclonal rabbit antiserum. Anti-testosterone was provided by Dr. Picaper (médecin nucléaire, CHU La Source, Orléans, France), anti-corticosterone antiserum was supplied by Merck. The bound fraction was then separated from free fraction by addition of dextran-coated charcoal and activity was counted on a tri-carb 2810 TR scintillation counter (Perkin Elmer, US). Androstenedione, in 2017 samples, was assayed with an ELISA kit supplied by IBL International. Some tests were performed to validate the hormone assays on egg yolk samples. Yolk extracts were serially diluted in the assay buffer and their displacement curves were parallel to the standard curve, for the three steroids. Inter- and intra-assay variations were respectively 12.43% and 5.80% for testosterone, 12.53% and 10.84% for corticosterone, and 7.27% and 8.70% for androstenedione. Testosterone, corticosterone, and androstenedione lowest detectable concentrations in yolk extracts were respectively 50.5 pg/mL, 56.3 pg/mL and 40 pg/mL. The assay specificity was evaluated by spiking extracts with the three steroids: recovery was 97.8% for testosterone, 113.3% for corticosterone and 100.67% for androstenedione.

Cross-reactions of testosterone antiserum were as follows : androsterone (63%), progesterone (1.45%), 17-β-estradiol (0.176%), corticosterone (0.41%), estrone (0.03%), aldosterone (<0.01%), cortisone (<0.01%). Cross-reactions of corticosterone antiserum were as follows : 11-dehydrocosticosterone (0.67%), deoxycorticosterone (1.5%), 18–hydroxy-deoxycorticosterone (<0.01%), cortisone (<0.01%), cortisone (<0.01%), progesterone (0.004%), aldosterone (0.2%). Cross-reactions of androstenedione antiserum were as follows: DHEA (1.8%), testosterone (0.20%), estrone (<0.1%), estradiol (<0.1%), progesterone (<0.1%), 17-OH-progesterone (<0.1%), 5α-dihydrotestosterone (<0.1%), cortisol (<0,01%) and DHEA-S (<0.01%).

**Table S 2.** Mean, range (min/max), standard deviation (sd) and sample sizes for each egg component measured in the eggs collected in 2014 and/or in 2017. The last row represents the sample size of each model, after excluding eggs with missing information for the response variable or covariates included in the models. Shell mass and albumen mass were measured but not included in the statistical analysis. The sample size of eggs analyzed for each component varied due to the capacity to assess some components only during one season (i.e.: lipids only in 2014 and vitamins and A4 only in 2017) or because the laboratory process failed for specific samples.

|  | Yolk mass  (g) | Egg mass  (g) | Shell mass  (g) | Albumen  mass  (g) | Lipids  (% yolk) | Proteins  (% yolk) | Carotenoids  (ug/g wet yolk) | Vitamin A  (ug/g wet yolk) | Vitamin E  (ug/g wet yolk) | Testosterone  (pg/mg wet yolk) | A4  (pg/mg wet yolk) | Corticosterone  (pg/mg wet yolk) |
| --- | --- | --- | --- | --- | --- | --- | --- | --- | --- | --- | --- | --- |
| **Mean** | 0.64 | 2.51 | 0.19 | 1.69 | 53.35 | 33.94 | 43.59 | 2.94 | 51.86 | 3.64 | 1.84 | 3.2 |
| **Min** | 0.37 | 2.04 | 0.12 | 0.97 | 9 | 28.95 | 9.65 | 0 | 4.14 | 1.35 | 1.2 | 0.01 |
| **Max** | 0.98 | 3.16 | 0.71 | 2.36 | 93.68 | 46.31 | 118.21 | 4.43 | 75.46 | 8.3 | 5 | 9.82 |
| **SD** | 0.1 | 0.2 | 0.09 | 0.23 | 15.13 | 2.33 | 22.03 | 0.98 | 13.26 | 1.12 | 0.73 | 1.3 |
| **N eggs** | 167 | 170 | 169 | 163 | 122 | 162 | 170 | 45 | 45 | 170 | 45 | 170 |
| **N clutches** | 59 | 58 | 59 | 58 | 43 | 57 | 59 | 16 | 16 | 59 | 16 | 59 |
| **N colonies** | 8 | 8 | 8 | 8 | 7 | 8 | 8 | 4 | 4 | 8 | 4 | 8 |
| **N seasons** | 2 | 2 | 2 | 2 | 1 | 2 | 2 | 1 | 1 | 2 | 1 | 2 |
| **N eggs model** | 122 | - | - | - | 83 | 117 | 119 | 36 | 36 | 122 | 36 | 122 |


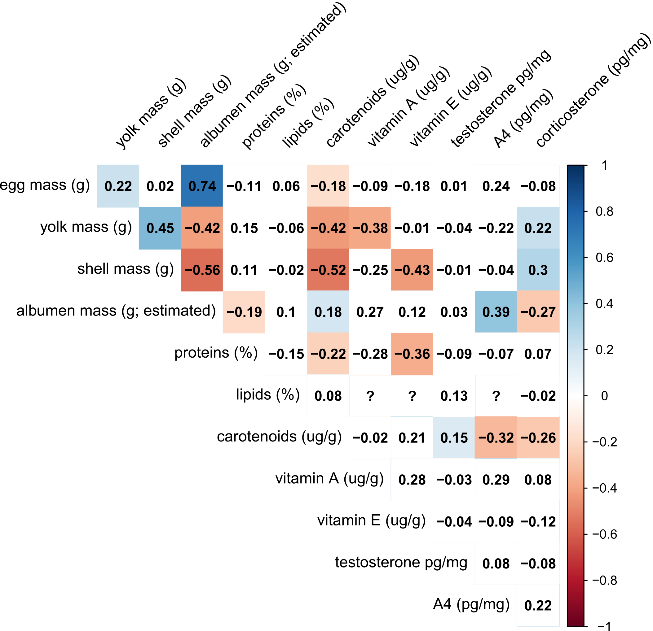

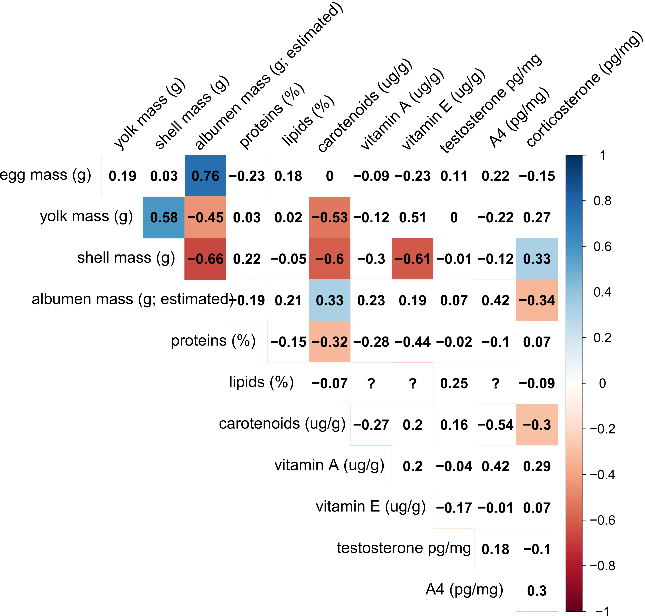


**Figure S 1.** Pearson correlation coefficients between egg components at the egg-level on the left (N=174) and at the clutch-level (mean per clutch) on the right (N=59), estimated using the Hmisc package (Harrell 2020). Blue squares represent positive correlations and red squares represent negative correlations with p<0.05. White squares represent non-significant correlations. Correlations that could not be estimated are represented by a question mark (when two components were not assessed in the same egg/clutch). The strongest correlation among components used as response variables (see Statistical analyses) was between yolk mass and yolk carotenoids concentration (cor=-0.42; p<0.001). No clear correlations were detected between hormones. Hormones showed significant, but weak (and mainly negative) correlations with carotenoids concentration. There are no qualitative differences between the correlations at the egg and at the clutch levels.

### Identification of breeding females

The sex of the birds seen visiting the nests was genetically determined from blood samples (Paquet, Doutrelant, et al. 2015). Breeders of each nest were genetically identified using full-likelihood parentage inference (Paquet, Doutrelant, et al. 2015; Fortuna et al. 2021). When no genetic data was available, parentage was determined based on the birds’ biology (e.g., age or pedigree; for details on parentage attribution see Supplementary materials in Fortuna et al., 2021).

### Group size of females from collected clutches

Group size of females whose clutches had been collected was expected to be similar in their next breeding attempt, as no juveniles had been produced and most replacement clutches were laid within 2 months’ time. In any case, we used a long-term database to test if the group size of breeding females in two consecutive breeding attempts was correlated. For this, we estimated the correlation between groups of the same breeding females in consecutive breeding attempts separated in time by a maximum of 3 months, as only 4 out of the 51 following breeding attempts of females with collected clutches had a difference above 3 months. We only compared nests with their next breeding attempt when no chicks fledged in the first attempt, and therefore no juveniles became part of the group. Mean group size was calculated as described in the main text and the Spearman’s rank correlation coefficient was assessed using the package ‘RVAideMemoire’ which estimates confidence intervals by bootstrapping (we used default number of replicates; Hervé 2021). We found a mean correlation of 0.57 (95CI=[0.33;0.75]; p<0.001; N=50) between the size of two consecutive groups of the same breeding female. We thus considered that this estimation of a moderate to high correlation between group sizes of consecutive breeding attempts, and the previous knowledge of the system on the composition of the groups (i.e., mostly offspring of the breeding pair), validated the use of this variable as a proxy of the breeding females group size at the time they laid the collected clutches.

### Statistical analyses

#### Fledging probability model

To test how fledging probability varied with laying order, and whether first and last eggs were more and less likely to fledge, respectively, we fitted three binomial general linear mixed models (GLMM) with ‘fledged’ as a binary response variable (0 if the chick did not fledge, 1 if it did) and, in each separate model, either laying order (as a continuous variable), or first egg or last egg (both as categorical variables scored as 0/1) as variables of interest (the latter results were qualitatively similar and were therefore omitted from the results, but analyses can be replicated using the code provided in <https://osf.io/raupk/?view_only=4283a309450948b88b3ba729f2362310>). We controlled for clutch size and egg mass as fixed effects and fitted nest identity nested in breeding female identity as random terms, as well as colony identity and season, to account for non-independence. This GLMM was fitted using the lme4 package (Bates et al. 2015). Numerical independent variables were centered and scaled by subtracting their mean and dividing by one standard deviation (Schielzeth 2010). Spearman rank correlation coefficients between variables were never above 0.31. We report effects as statistically significant when 95% confidence intervals (95CI) do not overlap zero. Plots show raw data and the predicted effects were estimated using the ggeffects package (Lüdecke 2018). To decide which variable to use (laying order, first egg or last egg) to test how maternal allocation varies with egg position in the laying sequence, we compared the three fledging probability models using Akaike Information Criteria (AIC) scores and used the variable included in the model with lowest AIC (‘laying order’, see below).

#### Egg mass, yolk mass and contents models

Since ‘last egg’ and ‘laying order’ were equally supported in the fledging probability analysis (see above), for each dependent variable we fitted one model with laying order as a continuous variable and a second model replacing laying order by the ‘last egg’ binary variable (1 if it was the last egg, 0 if not). Methods to run both models are available in the code provided, but only laying order models are presented below and in the main text, after confirming that in most cases these had higher R^2^ and lower DIC (deviance information criterion; see Tables S4-S13).

For all models, numerical independent variables were scaled and centered as previously described and numerical response variables were scaled by dividing by one standard deviation. Models of egg mass and contents were fitted assuming a normal error distribution, with default priors for fixed effects and vague priors for random terms and the residuals, with the degree-of-belief parameter (nu) set to 0.002 and variance (V) set to 1. We assessed residuals’ normal distribution and deviation from posterior predicted values based on 1000 model simulations using the DHARMa package (Hartig 2021). Three chains were run, and model convergence was assessed visually through trace plots and by calculating Gelman–Rubin statistic (all values <1.1; Gelman & Rubin, 1992).

For each chain of the egg mass model, 200,000 iterations were run, with samples taken every 195 iterations and the first 5,000 removed as burn-in, resulting in 1000 samples and ensuring low autocorrelation among thinned samples (<0.1). Effective sample size was ≥1000 for all parameters.

For egg content variables, when data distribution seemed closer to a normal distribution when on the log scale (only the case for A4), that response variable was fitted as well using log scale and the normality of the residuals of the two models was visually compared prior to looking at the results. The model with a residuals’ distribution closer to normal was used for inference.

Since yolk and egg mass vary with laying order in this species (van Dijk et al. 2013; this study, see below), each model was also fitted including yolk mass and egg mass as covariates (only yolk mass for vitamins and A4 models). For the response variables that represent concentrations (all but yolk mass), this allowed to test how the absolute level of a component varied with laying order and group size. For the yolk mass model, including egg mass allowed to test how the proportion of yolk mass relative to egg mass correlated with the variables of interest, and excluding it showed effects on the absolute yolk mass of the eggs. Plots show results on the relative amount of each egg component. Results on absolute terms are described in the Results when the effects of the variables of interest differed from the model on relative terms, and all results can be found below (see Results).

Regarding correlations between variables included in the models, absolute values of Spearman rank correlation coefficients between predation treatment and season were high in some datasets (up to 0.59). Similarly, wet yolk mass and season were correlated (up to 0.56) possibly due to differences in defrosting stage, as more time was needed to weigh the samples in 2014 (larger sample size). In the lipids’ dataset, clutch size and predation treatment showed a strong correlation (-0.54). However, these covariates were kept in the models since these were not variables of interest and a correlated variable may still explain some additional variation. All remaining correlation coefficients were below 0.42.

Egg content models were run following the same steps and using the same priors as described before, but number of iterations, burn-in (always ≥ 5,000) and thinning intervals were set to higher values in some models to ensure an effective sample size ≥1000 for all parameters (see code for details).

For all models, we present scaled coefficients of numerical variables, together with marginal and conditional R^2^ (variance explained only by fixed effects and by both fixed and random effects, respectively), calculated using code adapted from Nakagawa and Schielzeth (2013), and pMCMC (p). Plots show raw data and the predicted effects estimated using the ggeffects package (Lüdecke 2018).

## **Results**

### Fledging probability

**Table S 3.** Standardized estimates of the models testing the effect of egg position in the laying sequence on **fledging probability** (N=419). Results from a binomial GLMMs including ‘laying order’, as variable of interest. Statistically supported effects are presented in bold. Random effects variance, AIC and R^2^ (Nakagawa and Schielzeth 2013) are also presented.

|  |  | | |  | | |  | | |
| --- | --- | --- | --- | --- | --- | --- | --- | --- | --- |
| **Fixed effect** | **Log-Odds** | **95CI** | **p** |  |  |  |  |  |  |
| Fledged (Intercept) | -0.04 | -1.11  1.04 | 0.947 |  |  |  |  |  |  |
| **Laying order** | **-0.58** | **-0.95  -0.21** | **0.002** |  |  |  |  |  |  |
| Clutch size | -0.24 | -0.71  0.23 | 0.322 |  |  |  |  |  |  |
| Egg mass | 0.01 | -0.40  0.42 | 0.950 |  |  |  |  |  |  |
| **Random Effect** | | | | | | | | | |
| Mother ID:Nest ID (N=258) | 1.93 | | |  | | |  | | |
| Mother ID (N=176) | 2.06 | | |  | | |  | | |
| Colony ID (N=16) | 0.57 | | |  | | |  | | |
| Season (N=8) | 1.37 | | |  | | |  | | |
| Residuals | 3.29 | | |  | | |  | | |
| AIC | 526.3 | | |  | | |  | | |
| Marginal R^2^ / Conditional R^2^ | 0.05 / 0.661 | | |  | | |  | | |

### Egg mass

**Table S 4.** Standardized estimates of the models with **egg mass** as response variable (N=779). Posterior means and 95CrI for each variable are shown, as well as pMCMC (p) when applicable (all effective sample sizes ≥ 1000). Model marginal and conditional R^2^ (R^2^m and R^2^c, respectively) and DIC are given below. Statistically supported effects are presented in bold. The response variable was scaled before model fitting (SD= 0.20).

|  |  | | | |  | | | |
| --- | --- | --- | --- | --- | --- | --- | --- | --- |
| **Fixed effect** | **Post. mean** | **95CrI** | | **p** |  |  | |  |
| Intercept | 12.67 | 12.49 | 12.83 | 0.001 |  |  |  |  |
| Group size:laying order | -0.01 | -0.05 | 0.04 | 0.602 |  |  |  |  |
| Group size | -0.06 | -0.15 | 0.02 | 0.210 |  |  |  |  |
| **Laying order** | **0.19** | **0.14** | **0.23** | **0.001** |  |  |  |  |
| Clutch size | -0.07 | -0.15 | 0.02 | 0.102 |  |  |  |  |
| Tarsus size | 0.07 | -0.05 | 0.18 | 0.268 |  |  |  |  |
| **Random effect** |  |  |  |  |  |  |  |  |
| Mother ID:Nest ID  N=326 | 0.16 | 0.08 | 0.24 |  |  |  |  |  |
| Mother ID  N=192 | 0.47 | 0.34 | 0.62 |  |  |  |  |  |
| Colony ID  N=14 | 0.01 | 0 | 0.04 |  |  |  |  |  |
| Season  N=7 | 0.01 | 0 | 0.05 |  |  |  |  |  |
| Residuals | 0.33 | 0.29 | 0.37 |  |  |  |  |  |
| R^2^ m | 0.05 | 0.02 | 0.07 |  |  |  |  |  |
| R^2^ c | 0.70 | 0.56 | 0.84 |  |  |  |  |  |
| DIC | 1566.4 |  |  |  |  |  |  |  |


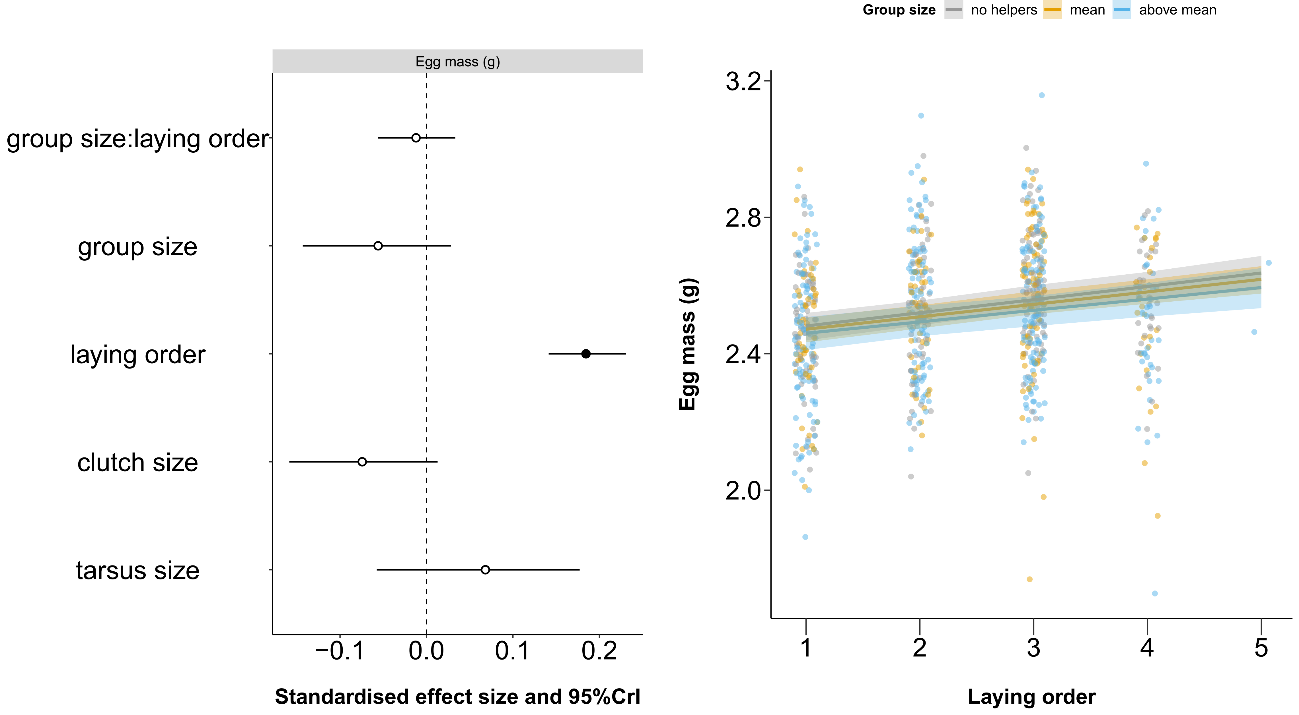


**Figure S 2.** On the left, egg mass model standardized coefficients and 95CrI. On the right, predicted relationship between egg mass and laying order for females with different group sizes. Lines represent the predicted values for the correlation between egg mass and laying order for three group size values: group size=2 (no helpers), mean group size (3.5) and the average between mean and maximum group size (5.5). Points represent raw data and point colors represent observations for groups without helpers, groups between group size=2 and mean group size or group sizes above the mean (all values rounded to the nearest integer).

### Egg contents

#### Yolk mass, lipids and proteins

**Table S 5.** Standardized estimates of the models with **yolk mass** (g) as response variable (N=122). Posterior means and 95CrI for each variable are shown, as well as pMCMC (p) when applicable (all effective sample sizes ≥ 1000). Model marginal and conditional R^2^ (R^2^m and R^2^c, respectively) and DIC are given below. Statistically supported effects are presented in bold. The response variable was scaled before model fitting (SD= 0.10). Reference level (intercept) for ‘protected’ and ‘season’ is 0 (not protected and 2014/2015, respectively).

|  | **Model with egg mass**  **(change in proportion of yolk)** | | | | | | | | **Model without egg mass**  **(absolute change)** | | | | | | |  |
| --- | --- | --- | --- | --- | --- | --- | --- | --- | --- | --- | --- | --- | --- | --- | --- | --- |
| **Fixed effect** | **Post. mean** | | **95CrI** | | | **p** | | | **Post. mean** | | **95CrI** | | | **p** | | |
| Intercept | 5.38 | | 4.92 | | 5.91 | | 0.001 | | 5.42 | | 4.9 | | 6.01 | 0.001 | | |
| **GS:laying order** | **0.12** | | **0.02** | | **0.24** | | **0.034** | | **0.11** | | **0.01** | | **0.24** | **0.068** | | |
| Group size | 0.11 | | -0.07 | | 0.30 | | 0.276 | | 0.11 | | -0.09 | | 0.32 | 0.304 | | |
| Laying order | **-0.16** | | **-0.27** | | **-0.04** | | **0.004** | | -0.12 | | -0.23 | | 0.01 | 0.064 | | |
| **Egg mass** | **0.29** | | **0.14** | | **0.44** | | **0.001** | | **-** | | **-** | | **-** | **-** | | |
| Clutch size | -0.03 | | -0.20 | | 0.14 | | 0.678 | | -0.08 | | -0.26 | | 0.10 | 0.350 | | |
| Protected 1 | 0.43 | | -0.10 | | 1.00 | | 0.126 | | 0.42 | | -0.22 | | 1.06 | 0.204 | | |
| **Season 1** | **1.69** | | **1.18** | | **2.23** | | **0.001** | | **1.56** | | **0.99** | | **2.11** | **0.001** | | |
| Colony ID N=7 | 0.10 | | 0 | | 0.38 | |  | | 0.09 | | 0 | | 0.39 |  | | |
| Mother ID N=42 | 0.13 | | 0 | | 0.29 | |  | | 0.18 | | 0 | | 0.35 |  | | |
| Residuals | 0.40 | | 0.27 | | 0.54 | |  | | 0.43 | | 0.30 | | 0.60 |  | | |
| R^2^ m | 0.46 | 0.29 | | 0.59 | | | |  | 0.39 | 0.28 | | 0.53 | | |  | |
| R^2^ c | 0.65 | 0.50 | | 0.79 | | | |  | 0.61 | 0.45 | | 0.77 | | |  | |
| DIC | 260.1 |  | |  | | | |  | 269.7 |  | |  | | |  | |

**Table S 6.** Standardized estimates of the models with **lipids** (%) as response variable (N=83). Posterior means and 95CrI for each variable are shown, as well as pMCMC (p) when applicable (all effective sample sizes ≥ 1000). Model marginal and conditional R^2^ (R^2^m and R^2^c, respectively) and DIC are given below. Statistically supported effects are presented in bold. The response variable was scaled before model fitting (SD=15.5). Reference level (intercept) for ‘protected’ is 0 (not protected).

|  | **Model without egg/yolk mass**  **(changes in concentration)** | | | | | | | | | **Model with egg/yolk mass**  **(absolute change)** | | | | | | | |
| --- | --- | --- | --- | --- | --- | --- | --- | --- | --- | --- | --- | --- | --- | --- | --- | --- | --- |
| **Fixed effect** | **Post. mean** | | **95CrI** | | | **p** | | | | **Post. mean** | | **95CrI** | | | **p** | | |
| Intercept | 3.38 | | 2.65 | | 4.11 | | 0.001 | | | 3.37 | | 2.63 | | 4.23 | 0.001 | | |
| **GS:laying order** | **0.21** | | **0.01** | | **0.39** | | **0.030** | | | 0.23 | | -0.01 | | 0.41 | 0.038 | | |
| Group size | **0.33** | | **0.1** | | **0.56** | | **0.010** | | | **0.34** | | **0.08** | | **0.55** | **0.012** | | |
| Laying order | -0.08 | | -0.28 | | 0.11 | | 0.446 | | | -0.09 | | -0.30 | | 0.14 | 0.398 | | |
| Yolk mass | **-** | | **-** | | **-** | | **-** | | | -0.07 | | -0.34 | | 0.17 | 0.606 | | |
| Egg mass | **-** | | **-** | | **-** | | **-** | | | 0.06 | | -0.17 | | 0.31 | 0.578 | | |
| Clutch size | 0.16 | | -0.09 | | 0.43 | | 0.222 | | | 0.15 | | -0.14 | | 0.42 | 0.306 | | |
| Protect. 1 | 0.14 | | -0.65 | | 1.03 | | 0.724 | | | 0.15 | | -0.80 | | 1.01 | 0.692 | | |
| **Random effect** | | | | | | | | |  | | | | | | | |  |
| Colony ID N=6 | 0.04 | | 0 | | 0.18 | |  | | | 0.04 | | 0 | | 0.19 |  | | |
| Mother ID N=28 | 0.03 | | 0 | | 0.11 | |  | | | 0.03 | | 0 | | 0.13 |  | | |
| Residuals | 0.84 | | 0.58 | | 1.12 | |  | | | 0.86 | | 0.61 | | 1.16 |  | | |
| R^2^ m | 0.23 | 0.10 | | 0.37 | | | |  | | 0.25 | 0.12 | | 0.39 | | |  | |
| R^2^ c | 0.28 | 0.12 | | 0.44 | | | |  | | 0.30 | 0.14 | | 0.46 | | |  | |
| DIC | 229.5 |  | |  | | | |  | | 233 |  | |  | | |  | |

**Table S 7.** Standardized estimates of the models with **proteins** (%) as response variable (N=117). Posterior means and 95CrI for each variable are shown, as well as pMCMC (p) when applicable (all effective sample sizes ≥ 1000). Model marginal and conditional R^2^ (R^2^m and R^2^c, respectively) and DIC are given below. Statistically supported effects are presented in bold. The response variable was scaled before model fitting (SD= 2.49). Reference level (intercept) for ’protected’ and ‘season’ is 0 (not protected and 2014/2015, respectively).

|  | **Model without egg/yolk mass**  **(changes in concentration)** | | | | | | | | | **Model with egg/yolk mass**  **(absolute change)** | | | | | | | |
| --- | --- | --- | --- | --- | --- | --- | --- | --- | --- | --- | --- | --- | --- | --- | --- | --- | --- |
| **Fixed effect** | **Post. mean** | | **95CrI** | | | **p** | | | | **Post. mean** | | **95CrI** | | | **p** | | |
| Intercept | 13.68 | | 13.09 | | 14.23 | | 0.001 | | | 13.86 | | 13.13 | | 14.47 | 0.001 | | |
| GS:laying order | 0.06 | | -0.13 | | 0.21 | | 0.504 | | | 0.04 | | -0.13 | | 0.21 | 0.652 | | |
| Group size | 0.07 | | -0.16 | | 0.32 | | 0.580 | | | 0.05 | | -0.23 | | 0.30 | 0.692 | | |
| Laying order | 0.07 | | -0.11 | | 0.24 | | 0.462 | | | 0.13 | | -0.06 | | 0.30 | 0.184 | | |
| Yolk mass | **-** | | **-** | | **-** | | **-** | | | 0.24 | | -0.03 | | 0.53 | 0.096 | | |
| Egg mass | **-** | | **-** | | **-** | | **-** | | | -0.21 | | -0.43 | | 0.01 | 0.074 | | |
| Clutch size | -0.05 | | -0.28 | | 0.18 | | 0.650 | | | -0.05 | | -0.30 | | 0.18 | 0.654 | | |
| Protect. 1 | -0.15 | | -0.8 | | 0.52 | | 0.614 | | | -0.23 | | -1.00 | | 0.37 | 0.496 | | |
| Season 1 | 0.25 | | -0.31 | | 0.86 | | 0.384 | | | -0.18 | | -0.99 | | 0.57 | 0.626 | | |
| **Random effect** | | | | | | | | |  | | | | | | | |  |
| Colony ID N=7 | 0.03 | | 0 | | 0.14 | |  | | | 0.04 | | 0 | | 0.15 |  | | |
| Mother ID N=40 | 0.11 | | 0 | | 0.36 | |  | | | 0.17 | | 0 | | 0.47 |  | | |
| Residuals | 0.94 | | 0.67 | | 1.26 | |  | | | 0.88 | | 0.62 | | 1.19 |  | | |
| R^2^ m | 0.08 | 0.01 | | 0.15 | | | |  | | 0.13 | 0.04 | | 0.23 | | |  | |
| R^2^ c | 0.20 | 0.02 | | 0.40 | | | |  | | 0.29 | 0.07 | | 0.52 | | |  | |
| DIC | 341.3 |  | |  | | | |  | | 337.6 |  | |  | | |  | |

#### Total carotenoids, vitamin A and vitamin E

**Table S 8.** Standardized estimates of the models with **total carotenoids** (ug/g) as response variable (N=119). Posterior means and 95CrI for each variable are shown, as well as pMCMC (p) when applicable (all effective sample sizes ≥ 1000). Model marginal and conditional R^2^ (R^2^m and R^2^c, respectively) and DIC are given below. Statistically supported effects are presented in bold. The response variable was scaled before model fitting (SD=20.7). Reference level (intercept) for ‘protected’ and ‘season’ is 0 (not protected and 2014/2015, respectively).

|  | **Model without egg/yolk mass**  **(changes in concentration)** | | | | | | | | | **Model with egg/yolk mass**  **(absolute change)** | | | | | | | |
| --- | --- | --- | --- | --- | --- | --- | --- | --- | --- | --- | --- | --- | --- | --- | --- | --- | --- |
| **Fixed effect** | **Post. mean** | | **95CrI** | | | **p** | | | | **Post. mean** | | **95CrI** | | | **p** | | |
| Intercept | 2.34 | | 1.68 | | 2.93 | | 0.001 | | | 2.22 | | 1.63 | | 2.91 | 0.001 | | |
| GS:laying order | -0.05 | | -0.14 | | 0.06 | | 0.350 | | | -0.03 | | -0.13 | | 0.06 | 0.522 | | |
| Group size | 0.03 | | -0.22 | | 0.28 | | 0.812 | | | 0.06 | | -0.16 | | 0.34 | 0.610 | | |
| **Laying order** | **-0.35** | | **-0.46** | | **-0.25** | | **0.001** | | | **-0.34** | | **-0.45** | | **-0.23** | **0.001** | | |
| Yolk mass | - | | - | | - | | - | | | -0.17 | | -0.35 | | -0.01 | 0.046 | | |
| **Egg mass** | **-** | | **-** | | **-** | | **-** | | | **-0.19** | | **-0.36** | | **-0.02** | **0.036** | | |
| Clutch size | 0.15 | | -0.04 | | 0.36 | | 0.150 | | | 0.13 | | -0.06 | | 0.34 | 0.216 | | |
| Protect. 1 | 0.04 | | -0.65 | | 0.66 | | 0.884 | | | 0.11 | | -0.60 | | 0.76 | 0.720 | | |
| **Season 1** | **-1.04** | | **-1.70** | | **-0.36** | | **0.004** | | | **-0.87** | | **-1.51** | | **-0.11** | **0.014** | | |
| **Random effect** | | | | | | | | |  | | | | | | | |  |
| Colony ID N=7 | 0.08 | | 0 | | 0.34 | |  | | | 0.09 | | 0 | | 0.38 |  | | |
| Mother ID N=41 | 0.33 | | 0.13 | | 0.55 | |  | | | 0.32 | | 0.13 | | 0.52 |  | | |
| Residuals | 0.32 | | 0.22 | | 0.42 | |  | | | 0.29 | | 0.20 | | 0.37 |  | | |
| R^2^ m | 0.38 | 0.23 | | 0.54 | | | |  | | 0.42 | 0.25 | | 0.57 | | |  | |
| R^2^ c | 0.72 | 0.60 | | 0.82 | | | |  | | 0.76 | 0.65 | | 0.85 | | |  | |
| DIC | 233.8 |  | |  | | | |  | | 222.9 |  | |  | | |  | |

**Table S 9**. Standardized estimates of the models with **vitamin A** (ug/g) as response variable (N=36). Posterior means and 95CrI for each variable are shown, as well as pMCMC (p) when applicable (all effective sample sizes ≥ 1000). Model marginal and conditional R^2^ (R^2^m and R^2^c, respectively) and DIC are given below. Statistically supported effects are presented in bold. The response variable was scaled before model fitting (SD=1.05).

|  | **Model without egg/yolk mass**  **(changes in concentration)** | | | | | | | | | **Model with egg/yolk mass**  **(absolute change)** | | | | | | | |
| --- | --- | --- | --- | --- | --- | --- | --- | --- | --- | --- | --- | --- | --- | --- | --- | --- | --- |
| **Fixed effect** | **Post. mean** | | **95CrI** | | | **p** | | | | **Post. mean** | | **95CrI** | | | **p** | | |
| Intercept | 2.69 | | 2.14 | | 3.29 | | 0.001 | | | 2.70 | | 2.14 | | 3.25 | 0.001 | | |
| GS:laying order | -0.13 | | -0.37 | | 0.12 | | 0.264 | | | -0.11 | | -0.29 | | 0.12 | 0.276 | | |
| Group size | -0.27 | | -0.78 | | 0.29 | | 0.256 | | | -0.23 | | -0.73 | | 0.37 | 0.354 | | |
| Laying order | 0.23 | | -0.02 | | 0.48 | | 0.070 | | | 0.07 | | -0.20 | | 0.30 | 0.552 | | |
| **Yolk mass** | **-** | | **-** | | **-** | | **-** | | | **-0.37** | | **-0.70** | | **-0.07** | **0.022** | | |
| **Random effect** | | | | | | | | |  | | | | | | | |  |
| Colony ID N=4 | 0.14 | | 0 | | 0.53 | |  | | | 0.16 | | 0 | | 0.64 |  | | |
| Mother ID N=14 | 0.63 | | 0 | | 1.51 | |  | | | 0.64 | | 0 | | 1.48 |  | | |
| Residuals | 0.53 | | 0.20 | | 0.94 | |  | | | 0.42 | | 0.19 | | 0.83 |  | | |
| R^2^ m | 0.16 | 0.01 | | 0.35 | | | |  | | 0.25 | 0.04 | | 0.46 | | |  | |
| R^2^ c | 0.61 | 0.23 | | 0.91 | | | |  | | 0.70 | 0.40 | | 0.94 | | |  | |
| DIC | 88.8 |  | |  | | | |  | | 81.7 |  | |  | | |  | |

**Table S 10.** Standardized estimates of the models with **vitamin E** (ug/g) as response variable (N=36). Posterior means and 95CrI for each variable are shown, as well as pMCMC (p) when applicable (all effective sample sizes ≥ 1000). Model marginal and conditional R^2^ (R^2^m and R^2^c, respectively) and DIC are given below. Statistically supported effects are presented in bold. The response variable was scaled before model fitting (SD=13.3).

|  | **Model without egg/yolk mass**  **(changes in concentration)** | | | | | | | | | **Model with egg/yolk mass**  **(absolute change)** | | | | | | | |
| --- | --- | --- | --- | --- | --- | --- | --- | --- | --- | --- | --- | --- | --- | --- | --- | --- | --- |
| **Fixed effect** | **Post. mean** | | **95CrI** | | | **p** | | | | **Post. mean** | | **95CrI** | | | **p** | | |
| Intercept | 3.70 | | 2.92 | | 4.47 | | 0.001 | | | 3.69 | | 2.81 | | 4.50 | 0.001 | | |
| GS:laying order | -0.16 | | -0.33 | | 0.04 | | 0.088 | | | -0.15 | | -0.32 | | 0.02 | 0.086 | | |
| Group size | 0 | | -0.79 | | 0.64 | | 0.974 | | | 0.04 | | -0.61 | | 0.85 | 0.936 | | |
| **Laying order** | **-0.30** | | **-0.48** | | **-0.10** | | **0.006** | | | **-0.42** | | **-0.61** | | **-0.22** | **0.001** | | |
| **Yolk mass** | **-** | | **-** | | **-** | | **-** | | | **-0.27** | | **-0.52** | | **-0.02** | **0.034** | | |
| **Random effect** | | | | | | | | |  | | | | | | | |  |
| Colony ID N=4 | 0.26 | | 0 | | 0.86 | |  | | | 0.37 | | 0 | | 1.18 |  | | |
| Mother ID N=14 | 1.38 | | 0.24 | | 3.05 | |  | | | 1.43 | | 0.32 | | 2.98 |  | | |
| Residuals | 0.31 | | 0.13 | | 0.55 | |  | | | 0.27 | | 0.13 | | 0.48 |  | | |
| R^2^ m | 0.12 | 0.01 | | 0.27 | | | |  | | 0.16 | 0.03 | | 0.31 | | |  | |
| R^2^ c | 0.83 | 0.62 | | 0.98 | | | |  | | 0.86 | 0.70 | | 0.98 | | |  | |
| DIC | 72.6 |  | |  | | | |  | | 68.06 |  | |  | | |  | |

#### Testosterone, A4 and corticosterone

**Table S 11.** Standardized estimates of the models with **testosterone** (pg/mg) as response variable (N=122). Posterior means and 95CrI for each variable are shown, as well as pMCMC (p) when applicable (all effective sample sizes ≥ 1000). Model marginal and conditional R^2^ (R^2^m and R^2^c, respectively) and DIC are given below. Statistically supported effects are presented in bold. The response variable was scaled before model fitting (SD=1.13). Reference level (intercept) for ‘protected’ and ‘season’ is 0 (not protected and 2014/2015, respectively).

|  | **Model without egg/yolk mass**  **(changes in concentration)** | | | | | | | | | **Model with egg/yolk mass**  **(absolute change)** | | | | | | | |
| --- | --- | --- | --- | --- | --- | --- | --- | --- | --- | --- | --- | --- | --- | --- | --- | --- | --- |
| **Fixed effect** | **Post. mean** | | **95CrI** | | | **p** | | | | **Post. mean** | | **95CrI** | | | **p** | | |
| Intercept | 3.17 | | 2.43 | | 3.93 | | 0.001 | | | 3.02 | | 2.27 | | 3.86 | 0.001 | | |
| GS:laying order | -0.03 | | -0.16 | | 0.08 | | 0.624 | | | -0.01 | | -0.13 | | 0.10 | 0.798 | | |
| Group size | 0.21 | | -0.09 | | 0.50 | | 0.146 | | | 0.23 | | -0.10 | | 0.52 | 0.132 | | |
| Laying order | -0.07 | | -0.20 | | 0.05 | | 0.262 | | | -0.09 | | -0.21 | | 0.04 | 0.166 | | |
| Yolk mass | **-** | | **-** | | **-** | | **-** | | | -0.17 | | -0.38 | | 0.04 | 0.112 | | |
| Egg mass | **-** | | **-** | | **-** | | **-** | | | 0.02 | | -0.24 | | 0.22 | 0.882 | | |
| Clutch size | -0.09 | | -0.35 | | 0.12 | | 0.412 | | | -0.10 | | -0.34 | | 0.12 | 0.442 | | |
| Protect. 1 | 0 | | -0.80 | | 0.79 | | 0.986 | | | 0.10 | | -0.81 | | 0.90 | 0.778 | | |
| Season 1 | -0.06 | | -0.84 | | 0.77 | | 0.848 | | | 0.21 | | -0.65 | | 1.07 | 0.628 | | |
| **Random effect** | | | | | | | | |  | | | | | | | |  |
| Colony ID N=7 | 0.19 | | 0.0005 | | 0.61 | |  | | | 0.21 | | 0 | | 0.73 |  | | |
| Mother ID N=42 | 0.48 | | 0.19 | | 0.82 | |  | | | 0.50 | | 0.17 | | 0.85 |  | | |
| Residuals | 0.46 | | 0.32 | | 0.59 | |  | | | 0.45 | | 0.31 | | 0.59 |  | | |
| R^2^ m | 0.11 | 0.01 | | 0.23 | | | |  | | 0.13 | 0.03 | | 0.25 | | |  | |
| R^2^ c | 0.62 | 0.44 | | 0.78 | | | |  | | 0.64 | 0.47 | | 0.82 | | |  | |
| DIC | 285.5 |  | |  | | | |  | | 285.1 |  | |  | | |  | |

**Table S 12.** Standardized estimates of the models with **A4** (pg/mg) as response variable (N=36). Posterior means and 95CrI for each variable are shown, as well as pMCMC (p) when applicable (all effective sample sizes ≥ 1000). Model marginal and conditional R^2^ (R^2^m and R^2^c, respectively) and DIC are given below. Statistically supported effects are presented in bold. The response variable was used in the log scale and was scaled before model fitting (SD log=0.33).

|  | **Model without yolk mass**  **(changes in concentration)** | | | | | | | | | **Model with yolk mass**  **(absolute change)** | | | | | | | |
| --- | --- | --- | --- | --- | --- | --- | --- | --- | --- | --- | --- | --- | --- | --- | --- | --- | --- |
| **Fixed effect** | **Post. mean** | | **95CrI** | | | **p** | | | | **Post. mean** | | **95CrI** | | | **p** | | |
| Intercept | 1.72 | | 1.22 | | 2.29 | | 0.001 | | | 1.73 | | 1.20 | | 2.32 | 0.001 | | |
| GS:laying order | 0.03 | | -0.21 | | 0.28 | | 0.838 | | | 0.04 | | -0.20 | | 0.30 | 0.818 | | |
| Group size | -0.36 | | -0.78 | | 0.11 | | 0.098 | | | -0.35 | | -0.83 | | 0.04 | 0.120 | | |
| Laying order | 0.25 | | -0.001 | | 0.50 | | 0.056 | | | 0.22 | | -0.07 | | 0.50 | 0.142 | | |
| Yolk mass | **-** | | **-** | | **-** | | **-** | | | -0.04 | | -0.38 | | 0.29 | 0.796 | | |
| **Random effect** | | | | | | | | |  | | | | | | | |  |
| Colony ID N=4 | 0.19 | | 0 | | 0.58 | |  | | | 0.27 | | 0 | | 0.71 |  | | |
| Mother ID N=14 | 0.37 | | 0 | | 0.93 | |  | | | 0.39 | | 0 | | 1.05 |  | | |
| Residuals | 0.56 | | 0.24 | | 0.95 | |  | | | 0.58 | | 0.28 | | 1.02 |  | | |
| R^2^ m | 0.20 | 0.01 | | 0.40 | | | |  | | 0.21 | 0.02 | | 0.42 | | |  | |
| R^2^ c | 0.55 | 0.25 | | 0.87 | | | |  | | 0.56 | 0.24 | | 0.86 | | |  | |
| DIC | 91.1 |  | |  | | | |  | | 93.2 |  | |  | | |  | |

**Table S 13.** Standardized estimates of the models with **corticosterone** (pg/mg) as response variable (N=122). Posterior means and 95CrI for each variable are shown, as well as pMCMC (p) when applicable (all effective sample sizes ≥ 1000). Model marginal and conditional R^2^ (R^2^m and R^2^c, respectively) and DIC are given below. Statistically supported effects are presented in bold. The response variable was scaled before model fitting (SD=1.25). Reference level (intercept) for ‘protected’ and ‘season’ is 0 (not protected and 2014/2015, respectively).

|  | **Model without egg/yolk mass**  **(changes in concentration)** | | | | | | | | | **Model with egg/yolk mass**  **(absolute change)** | | | | | | | |
| --- | --- | --- | --- | --- | --- | --- | --- | --- | --- | --- | --- | --- | --- | --- | --- | --- | --- |
| **Fixed effect** | **Post. mean** | | **95CrI** | | | **p** | | | | **Post. mean** | | **95CrI** | | | **p** | | |
| Intercept | 2.54 | | 1.90 | | 3.19 | | 0.001 | | | 2.64 | | 1.93 | | 3.30 | 0.001 | | |
| GS:laying order | -0.02 | | -0.13 | | 0.11 | | 0.758 | | | -0.02 | | -0.14 | | 0.10 | 0.754 | | |
| Group size | -0.08 | | -0.34 | | 0.22 | | 0.584 | | | -0.08 | | -0.37 | | 0.22 | 0.574 | | |
| **Laying order** | **0.16** | | **0.01** | | **0.27** | | **0.014** | | | **0.18** | | **0.04** | | **0.31** | **0.004** | | |
| Yolk mass | **-** | | **-** | | **-** | | **-** | | | 0.12 | | -0.11 | | 0.30 | 0.278 | | |
| Egg mass | **-** | | **-** | | **-** | | **-** | | | -0.11 | | -0.31 | | 0.10 | 0.332 | | |
| Clutch size | 0.08 | | -0.16 | | 0.30 | | 0.512 | | | 0.07 | | -0.17 | | 0.28 | 0.546 | | |
| Protect. 1 | -0.12 | | -0.79 | | 0.66 | | 0.724 | | | -0.16 | | -0.86 | | 0.58 | 0.702 | | |
| Season 1 | 0.66 | | 0 | | 1.38 | | 0.056 | | | 0.44 | | -0.29 | | 1.26 | 0.286 | | |
| **Random effect** | | | | | | | | |  | | | | | | | |  |
| Colony ID N=7 | 0.04 | | 0 | | 0.16 | |  | | | 0.04 | | 0 | | 0.19 |  | | |
| Mother ID N=42 | 0.45 | | 0.16 | | 0.74 | |  | | | 0.47 | | 0.19 | | 0.82 |  | | |
| Residuals | 0.47 | | 0.33 | | 0.61 | |  | | | 0.47 | | 0.34 | | 0.64 |  | | |
| R^2^ m | 0.20 | 0.07 | | 0.34 | | | |  | | 0.21 | 0.08 | | 0.35 | | |  | |
| R^2^ c | 0.60 | 0.45 | | 0.77 | | | |  | | 0.62 | 0.46 | | 0.77 | | |  | |
| DIC | 286.7 |  | |  | | | |  | | 287.5 |  | |  | | |  | |


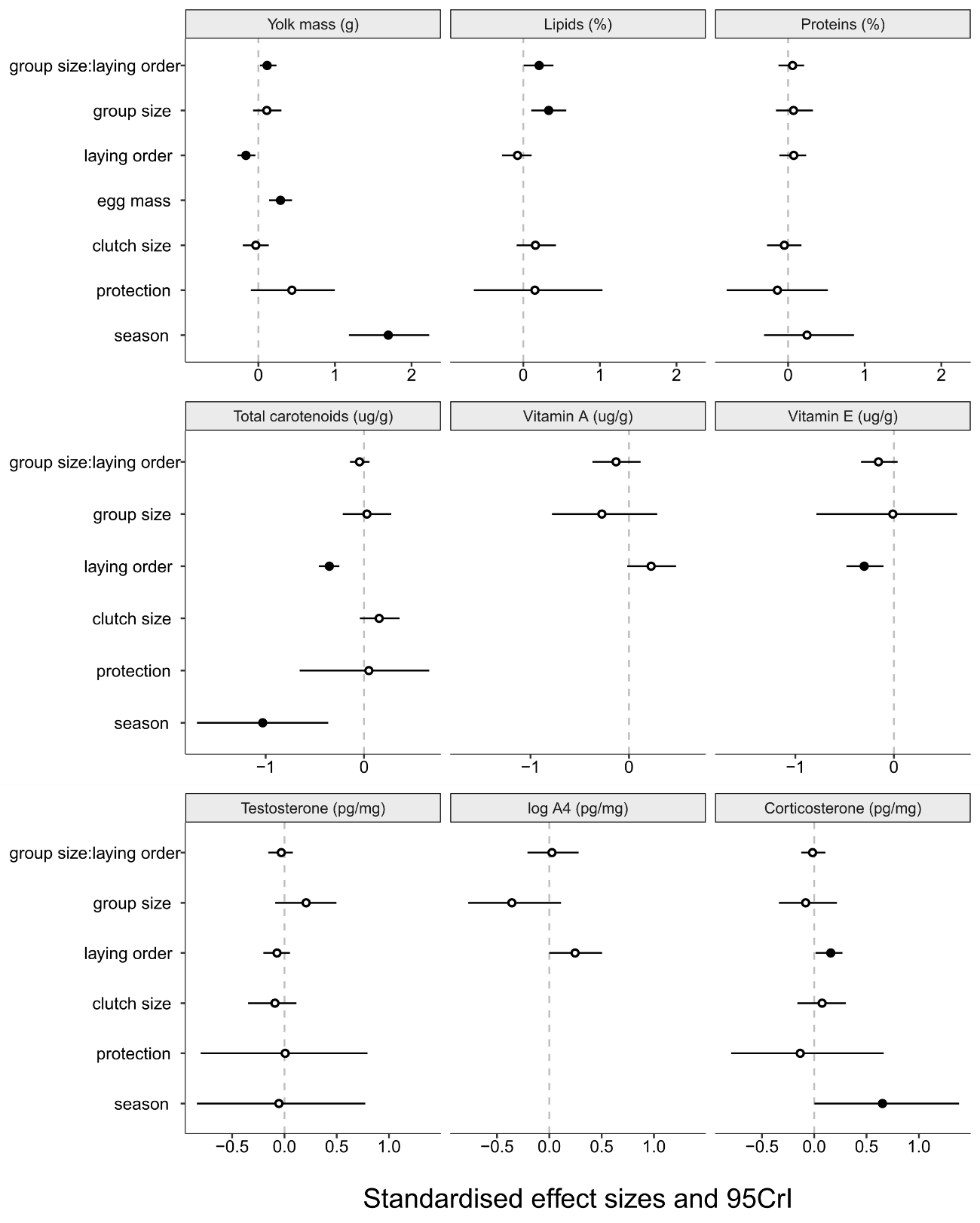


**Figure S 3.** Standardized posterior means represented by circles and 95CrI represented by bars of the variables of interest (interaction group size:laying order and its single terms) and covariates included in the model for each response variable. Filled circles show statistically credible effects.

### Helper effects on hatching and fledging success

**Table S 14.** Standardized posterior log odds’ means of the categorical GLMM testing the effect of the interaction between egg laying order and group size on **hatching probability** (N=331). Random effects posterior mean and 95CrI are also presented. Statistically supported effects are in bold.

|  |  | | | | |
| --- | --- | --- | --- | --- | --- |
| **Fixed effect** | **Log-Odds** | **95CrI** | | | **p** |
| Hatched (Intercept) | 1.06 | 0.66 | | 1.49 | 0.0005 |
| Group size:laying order | -0.08 | -0.41 | | 0.23 | 0.620 |
| Group size | 0.02 | -0.29 | | 0.36 | 0.880 |
| **Laying order** | **-0.74** | **-1.08** | | **-0.41** | **<0.001** |
| Clutch size | -0.04 | -0.39 | | 0.28 | 0.814 |
| **Egg mass** | **0.59** | **0.28** | | **0.94** | **0.001** |
| Mother ID:Nest ID (N=196) | 0.22 | 0 | 1.11 | |  |
| Mother ID (N=144) | 0.09 | 0 | 0.42 | |  |
| Colony ID (N=13) | 0.07 | 0 | 0.27 | |  |
| Season (N=7) | 0.08 | 0 | 0.35 | |  |
| Residuals | 1 | 1 | 1 | |  |


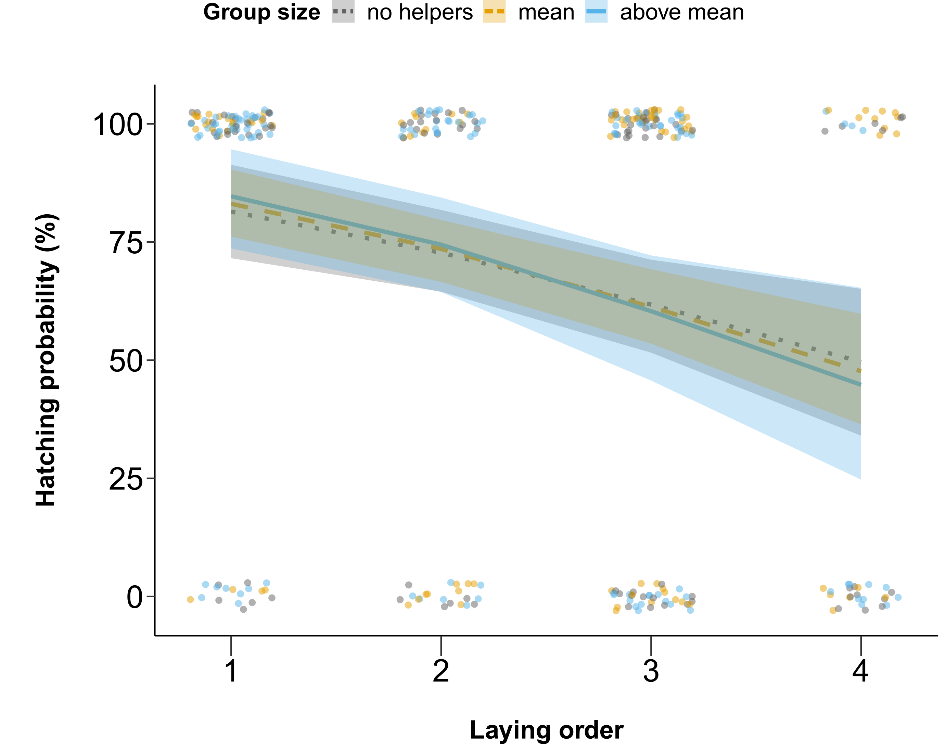


**Figure S 4.** Relationship between hatching probability and laying order for females of different group sizes. Lines represent the posterior predicted means and 95% credible intervals for the correlation between fledging probability and laying order for three group size values: group size=2 (no helpers; grey dotted line), mean group size (3.3; orange dashed line) and the average between mean and maximum group size (5.2; blue solid line). Points represent raw data and point colors represent observations for groups without helpers, groups between group size=2 and mean group size or group sizes above the mean (all values rounded to the nearest integer).

**Table S 15.** Standardized posterior log odds’ means of the categorical GLMM testing the effect of the interaction between egg laying order and group size on **fledging probability** (N=226). Random effects posterior mean and 95CrI are also presented. Statistically supported effects are in bold.

|  |  | | | | |
| --- | --- | --- | --- | --- | --- |
| **Fixed effect** | **Log-Odds** | **95CrI** | | | **p** |
| Fledged (Intercept) | 0.83 | -1.44 | | 3.64 | 0.428 |
| Group size:laying order | 0.18 | -0.59 | | 0.93 | 0.649 |
| **Group size** | **1.56** | **0.41** | | **2.79** | **0.002** |
| Laying order | -0.63 | -1.29 | | 0 | 0.047 |
| Clutch size | -0.69 | -1.78 | | 0.25 | 0.144 |
| Egg mass | -0.32 | -1.18 | | 0.44 | 0.425 |
| Mother ID:Nest ID (N=150) | 11.11 | 0 | 26.88 | |  |
| Mother ID (N=120) | 1.64 | 0 | 7.52 | |  |
| Colony ID (N=13) | 0.97 | 0 | 4.43 | |  |
| Season (N=7) | 7.91 | 0 | 26.01 | |  |
| Residuals | 1 | 1 | 1 | |  |

## **References**

Biard C, Gil D, Karadaş F, Saino N, Spottiswoode CN, Surai PF, Møller AP. 2009. Maternal Effects Mediated by Antioxidants and the Evolution of Carotenoid‐Based Signals in Birds. The American Naturalist. 174(5):696–708. doi:10.1086/606021.

Biard C, Surai PF, Møller AP. 2005. Effects of carotenoid availability during laying on reproduction in the blue tit. Oecologia. 144(1):32–44. doi:10.1007/s00442-005-0048-x.

Bligh EG, Dyer WJ. 1959. A rapid method of total lipid extraction and purification. Can J Biochem Physiol. 37(8):911–917. doi:10.1139/o59-099.

Covas R. 2002. Life-history evolution and cooperative breeding in the sociable weaver.

van Dijk RE, Eising CM, Merrill RM, Karadas F, Hatchwell B, Spottiswoode CN. 2013. Maternal effects in the highly communal sociable weaver may exacerbate brood reduction and prepare offspring for a competitive social environment. Oecologia. 171(2):379–389. doi:10.1007/s00442-012-2439-0.

Fortuna R, Paquet M, Ferreira AC, Silva LR, Theron F, Doutrelant C, Covas R. 2021. Maternal allocation in relation to weather, predation and social factors in a colonial cooperative bird. Journal of Animal Ecology. 90(5):1122–1133. doi:https://doi.org/10.1111/1365-2656.13438.

Harrell FE. 2020. Hmisc: harrell miscellaneous. R package version 4.4-2. R Found Stat Comput https://CRAN R-project org/package= Hmisc (accessed 16 Feb 2018).

Hartig F. 2021. DHARMa: residual diagnostics for hierarchical (multi-level/mixed) regression models. R package version 041. 4.

Hervé M. 2021. RVAideMemoire: testing and plotting procedures for biostatistics. R package version 09–79.

Lüdecke D. 2018. ggeffects: Tidy data frames of marginal effects from regression models. Journal of Open Source Software. 3(26):772.

Nakagawa S, Schielzeth H. 2013. A general and simple method for obtaining R2 from generalized linear mixed-effects models. Methods in Ecology and Evolution. 4(2):133–142. doi:https://doi.org/10.1111/j.2041-210x.2012.00261.x.

Paquet M, Covas R, Chastel O, Parenteau C, Doutrelant C. 2013. Maternal Effects in Relation to Helper Presence in the Cooperatively Breeding Sociable Weaver. PLoS ONE. 8(3). doi:10.1371/journal.pone.0059336.
